# Algorithms on the rise: a machine learning–driven survey of prostate cancer literature

**DOI:** 10.3389/fonc.2025.1675459

**Published:** 2025-10-02

**Authors:** Simin Gu, Jiajun Chen, Chunyan Fan, Xiaodong Huang, Linbo Li, Hua Zhang

**Affiliations:** ^1^ Department of Urology, Qidong People’s Hospital, Qidong Liver Cancer Institute, Affiliated Qidong Hospital of Nantong University, Qidong, Jiangsu, China; ^2^ Central Laboratory, Qidong People’s Hospital, Qidong Liver Cancer Institute, Affiliated Qidong Hospital of Nantong University, Qidong, Jiangsu, China

**Keywords:** prostate cancer, machine learning, bibliometric analysis, deep learning, radiomics, translational research

## Abstract

**Introduction:**

Machine learning (ML) has shown significant potential in improving prostate cancer (PCa) diagnosis, prognosis, and treatment planning. Despite rapid advancements, a comprehensive quantitative synthesis of global research trends and the knowledge structure of ML applications in PCa remains lacking. This study aimed to systematically map the evolution, research hotspots, and collaborative landscape of ML-PCa research.

**Methods:**

A systematic bibliometric review was performed on English-language articles and reviews published between January 2005 and December 2024. Publications were retrieved from the Web of Science (WOS) and Scopus databases. Analytical tools including CiteSpace, VOSviewer, and the R-bibliometrix package were employed to assess publication growth trends, country and institutional contributions, collaboration networks, author productivity, journal outlets, and keyword co-occurrence patterns.

**Results:**

A total of 2,632 publications were identified. Annual output increased from fewer than 20 papers during 2005–2014 to 661 in 2024, with 82% of all studies published since 2021. Emerging frontiers included deep learning, radiomics, and multimodal data fusion. China (649 publications) and the United States (492 publications) led in research volume, while Germany demonstrated the highest proportion of multinational collaboration (39.29%). Leading institutions by output were the Chinese Academy of Sciences, the University of British Columbia, and Shanghai Jiao Tong University. In terms of citation impact, the University of Toronto, Case Western Reserve University, and the University of Pennsylvania ranked highest. The journals Cancers, Frontiers in Oncology, and Scientific Reports published the most ML-PCa studies, highlighting the cross-disciplinary nature of the field. Madabhushi Anant emerged as the most central author hub in global collaboration networks.

**Discussion:**

ML applications in PCa research have experienced exponential growth, with methodological innovations driving interest in deep learning and radiomics. However, a persistent translational gap exists between algorithmic development and clinical implementation. Future directions should focus on fostering interdisciplinary collaboration, conducting prospective multicenter validation studies, and aligning with regulatory standards to accelerate the integration of ML models into clinical PCa workflows.

## Introduction

In recent years, prostate cancer (PCa) has emerged as a leading public-health challenge for men (1), with new cases accounting for approximately 14.1% of all male cancers and PCa-specific deaths comprising about 7% of global cancer mortality (2). Although the widespread adoption of prostate-specific antigen (PSA) screening since the 1990s and advances in surgical and radiotherapeutic techniques have improved early detection and treatment (3, 4), existing biomarkers still suffer from limited specificity, contributing to overdiagnosis rates as high as 30%–40% (5, 6). Moreover, high-risk subtypes such as castration-resistant PCa continue to bear poor prognoses, with five-year survival rates below 30% (7), underscoring the urgent need to transcend traditional diagnostic and therapeutic paradigms.

Machine learning (ML), as a transformative technological force, has demonstrated substantial promise across multiple facets of PCa management (8). In imaging diagnostics, MRI-based radiomics models and deep-learning algorithms have facilitated automated Gleason grading and early tumor detection, markedly enhancing diagnostic accuracy (9–11). In genomics, multimodal ML approaches can mine complex gene-expression profiles and exosomal signatures to uncover novel biomarkers (12, 13). At the therapeutic level, deep neural networks have been employed to predict patient outcomes under varying treatment regimens, guiding personalized medication strategies and radiotherapy planning (14, 15).

Despite these advances, concerns persist regarding reproducibility, external validation, and the clinical utility of ML applications. Multiple systematic reviews have highlighted a pattern of methodological innovation outpacing clinical readiness. For example, among AI systems benchmarked against clinicians (11, 16, 17), only a minority were prospectively tested or deployed in real-world settings, and adherence to reporting guidelines such as CONSORT-AI remains inconsistent (18). This imbalance underscores the need for bibliometric evaluations to characterize the trajectory of research outputs, identify key domains of progress, and expose areas where translational gaps remain.

Against this backdrop, the present study conducts a systematic review and bibliometric analysis of ML-PCa literature published between 2005 and 2024 in the Web of Science (WOS) and Scopus databases. Utilizing CiteSpace, VOSviewer, and the R-bibliometrix package, we analyze publication trends, authorship and institutional networks, journal and reference co-citations, and the evolution of thematic keywords, providing a structured overview of this rapidly evolving field. In addition to these descriptive analyses, we further examine proportional signals related to clinical validation and translation. Specifically, within our corpus (2005–2024; n=2,632), the explicit use of the keyword “validation” accounted for only ~2.8% (73/2,632), and terms directly reflecting prospective evaluation, randomization, or real-world implementation were absent among the most frequent author keywords. By combining quantitative bibliometric mapping with critical appraisal of clinical integration, we aim to pinpoint both the technological and implementation gaps that must be bridged for ML to fulfil its promise in PCa care.

## Methods

### Data collection and preprocessing

**Figure 1:**
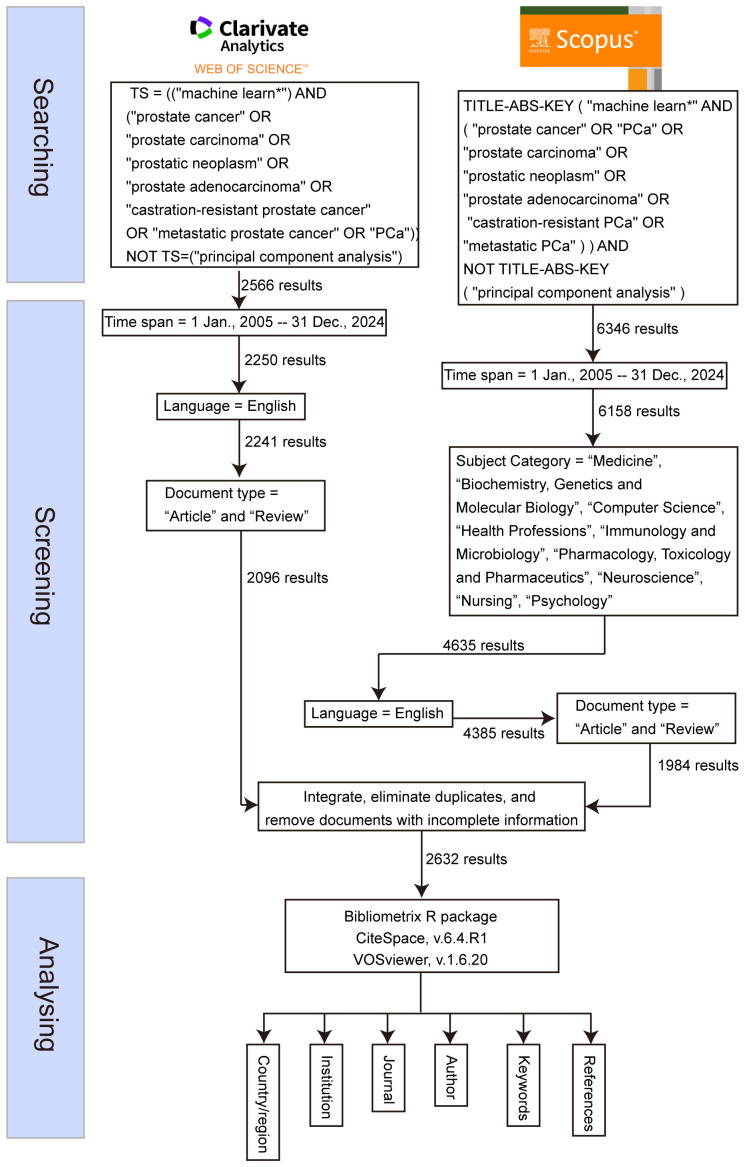
Flowchart of this study.

On July 5, 2025, a systematic literature search was conducted across two widely recognized databases: the WOS Core Collection and Scopus. The search strategy for WOS was defined by the query: TS=(“machine learn*”) AND (“prostate cancer” OR “prostate carcinoma” OR “prostatic neoplasm” OR “prostate adenocarcinoma” OR “castration-resistant prostate cancer” OR “metastatic prostate cancer” OR “PCa”), while the search for Scopus used the query: TITLE-ABS-KEY (“machine learn*” AND (“prostate cancer” OR “PCa” OR “prostate carcinoma” OR “prostatic neoplasm” OR “prostate adenocarcinoma” OR “castration-resistant PCa” OR “metastatic PCa”)). Both searches were limited to publications from January 1, 2005, to December 31, 2024, and restricted to articles and reviews published in English. For the Scopus database, additional filtering was applied to include only articles from the following subject categories: “Medicine,” “Biochemistry, Genetics and Molecular Biology,” “Computer Science,” “Health Professions,” “Immunology and Microbiology,” “Pharmacology, Toxicology and Pharmaceutics,” “Neuroscience,” “Nursing,” and “Psychology.” Following the retrieval, the documents from both databases were merged using Python (version 3.9.14), ensuring consistent terms across the two datasets. Duplicate entries were removed, and records with incomplete or missing information were excluded (**Figure 1**). The remaining documents were then retained for further analysis.

### Bibliometric toolchain configuration

The analysis leveraged a tripartite toolchain to ensure methodological rigor and multidimensional insights. First, CiteSpace 6.4.R1 (19, 20) was deployed to detect citation bursts and temporal trends. It was configured with 1-year time slices (2005–2024), a g-index term selection criterion (k=25), and Pathfinder network pruning (γ=0.7) to optimize cluster resolution. Concurrently, VOSviewer 1.6.20 (21) was used to generate co-authorship and keyword co-occurrence networks. For mapping countries and institutions, a minimum threshold of five documents per node was applied, with full counting and association strength normalization to minimize bias toward high-frequency terms. For statistical validation and thematic evolution tracking, bibliometrix (version 4.1.0) (22) in R (version 4.3.1) was used to perform Latent Dirichlet Allocation topic modeling—initiating 10 topics through Gibbs sampling over 2,000 iterations. Exponential smoothing (α=0.8) was applied to model productivity trends.

## Results

### Publication trends and document characteristics

**Figure 2:**
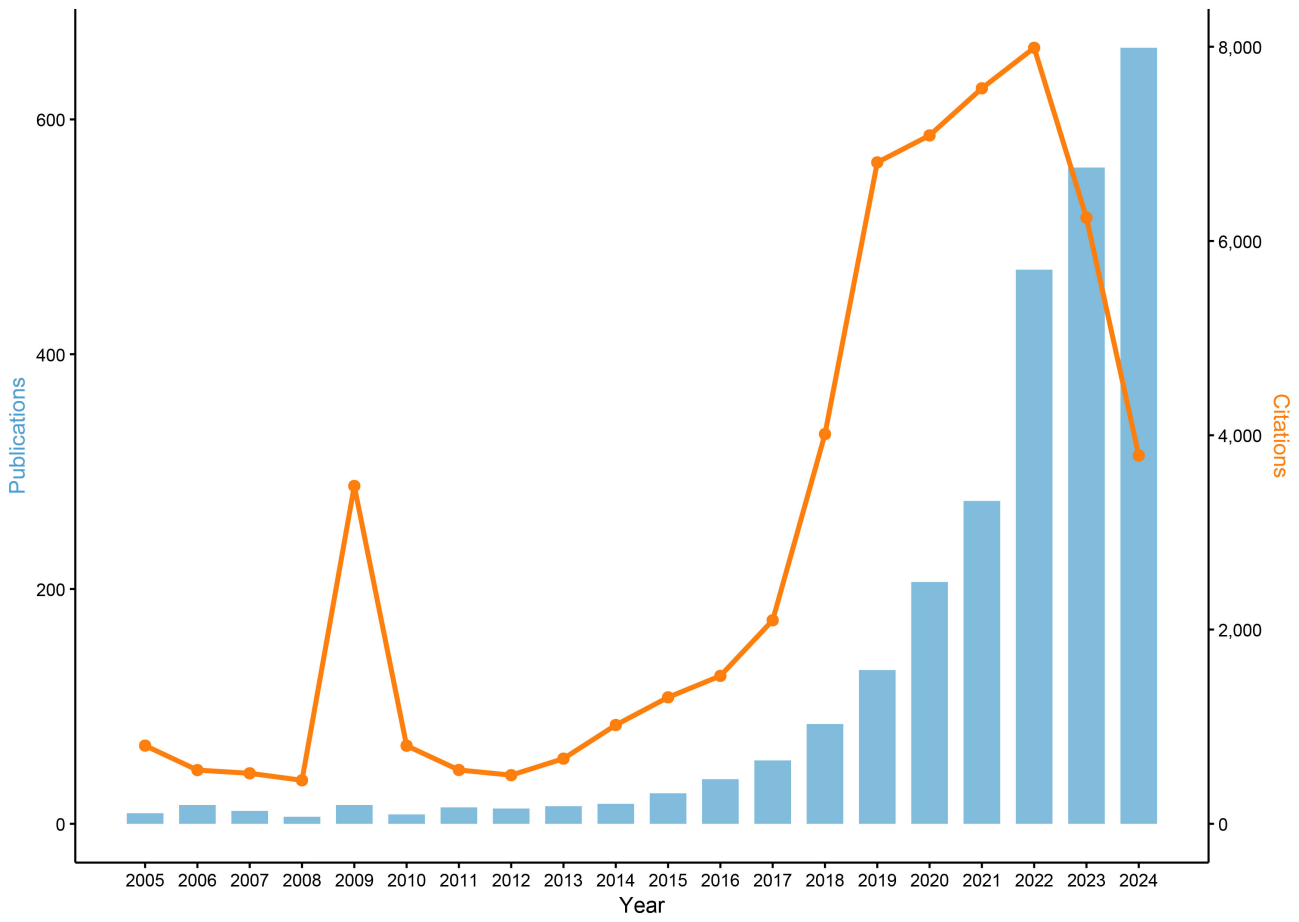
Annual trends in publications and citations related to ML-PCa research from 2005 to 2024.

Between 2005 and 2024, a comprehensive analysis identified 2,632 publications in the field of ML-PCa (**Figure 2**). Initial progress was measured, with annual publications consistently below 20 throughout 2005–2014. Accelerated growth commenced in 2015, driving a sustained increase in output that exceeded 100 articles by 2018 and reached 206 in 2020. The period 2021–2024 witnessed exponential expansion, with annual publications peaking at 472 (2022), 559 (2023), and 661 (2024). Collectively, this trajectory generated an average annual growth rate of 25.4% over the two-decade period, with 82% of cumulative publications (2,173/2,632) concentrated in the final four years (2021–2024). Cumulative citations reached 57,771, attesting to the field’s scholarly significance. Parallel citation trends show moderate fluctuations (400–800 citations annually) during 2005–2013, interrupted by a transient 2009 spike (3,478 citations) attributable to highly influential works. From 2014 onward, citations grew robustly, surpassing 4,000 in 2018 and surging to 6,809 in 2019. The 2020–2022 period sustained exceptional impact (7,000–8,000 citations annually), peaking at 7,989 in 2022. Although 2023–2024 saw a moderate decline to ~3,700 annual citations, persistently elevated levels confirm ML-PCa’s enduring academic relevance.

### Country contributions

**Figure 3:**
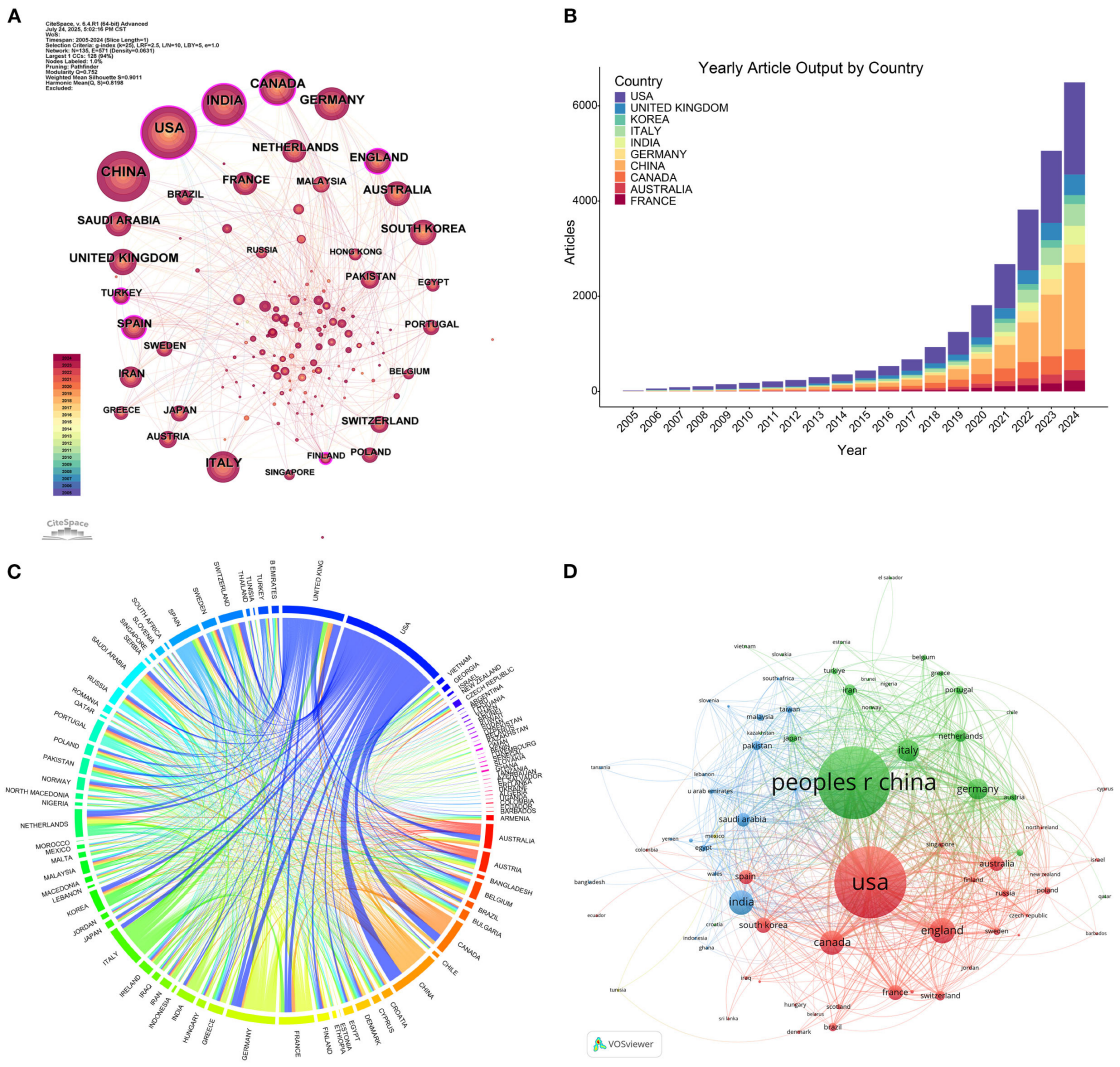
Country contributions and collaboration network in ML-PCa research. **(A)** Annual publication trends of the most productive countries from 2005 to 2024. **(B)** Global collaboration map illustrating international cooperation between countries or regions based on co-authorship. **(C)** Chord diagram of country-level collaborations, where the ribbon width represents the collaboration intensity between countries. **(D)** Country citation clustering network, where node size reflects the number of citations received by each country and node color indicates different citation clusters.

Globally, researchers from 92 countries/regions have contributed to ML-PCa research. China emerged as the dominant contributor with 649 publications (24.66% of total output), followed by the United States (492 publications, 18.69%) and India (162 publications, 6.16%) (**Table 1**). Network analysis positioned the United States and China as central hubs (**Figure 3A**), with the United Kingdom, Canada, and India forming key peripheral connections. Annual growth patterns indicate accelerated global output after 2018, with China and the United States establishing overwhelming dominance by 2024 (**Figure 3B**).

**Table 1:** Top 10 most productive countries in ML-PCa research and their pattern of international collaboration patterns.

Country	Articles	Articles %	SCP	MCP	MCP %
CHINA	649	24.66	561	88	13.56
USA	492	18.69	389	103	20.93
INDIA	162	6.16	144	18	11.11
UNITED KINGDOM	109	4.14	74	35	32.11
CANADA	108	4.10	76	32	29.63
ITALY	106	4.03	77	29	27.36
GERMANY	84	3.19	51	33	39.29
KOREA	72	2.74	53	19	26.39
AUSTRALIA	63	2.39	43	20	31.75
IRAN	50	1.90	38	12	24.00

Analysis of international collaboration revealed significant strategic differences: While China maintained a relatively low multinational collaboration proportion (MCP ratio=13.56%), the United States exhibited higher collaborative engagement (MCP ratio=20.93%). Germany demonstrated the most extensive international integration among top contributors (MCP ratio=39.29%) (**Table 1**).

Bilateral analysis identified the United States-China partnership (62 joint publications) as the strongest collaborative dyad, followed by United States-Canada (36) and United States-United Kingdom (34) (**Figure 3C**, **Table 2**). Citation-based clustering confirmed the dual centrality of the United States and China, while revealing distinct regional clusters: Italy, Germany, and the United Kingdom formed European-oriented groupings, whereas India anchored a separate citation community (**Figure 3D**).

**Table 2:** Top 10 bilateral collaborations in ML-PCa research.

From	To	Frequency
USA	CHINA	62
USA	CANADA	36
USA	UNITED KINGDOM	34
USA	GERMANY	26
ITALY	UNITED KINGDOM	20
USA	ITALY	19
UNITED KINGDOM	GERMANY	18
USA	FRANCE	18
USA	KOREA	16
CHINA	CANADA	15

### Institution features

**Figure 4:**
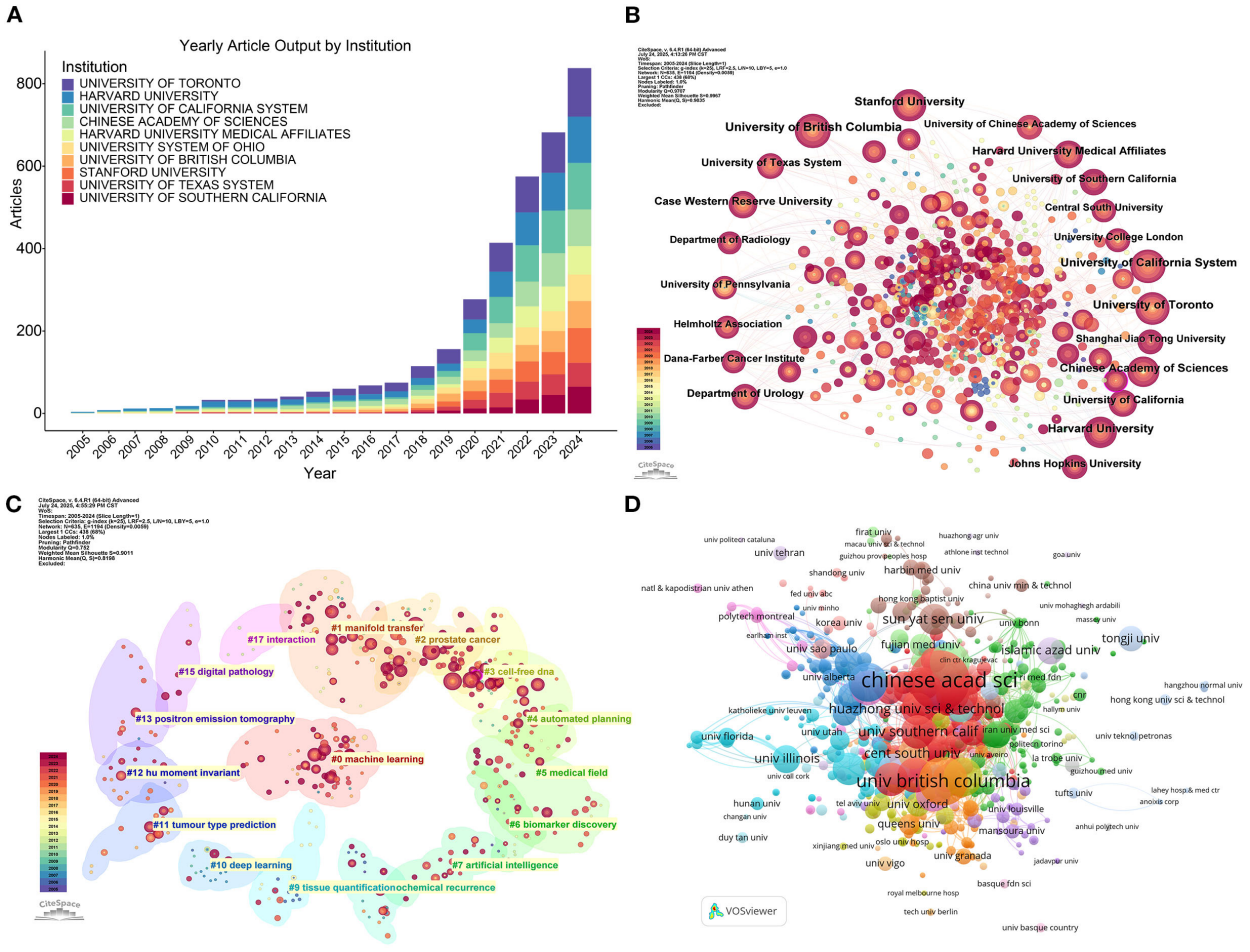
Institutional contributions to ML-PCa research. **(A)** Annual publication statistics of the top contributing institutions from 2005 to 2024. **(B)** Institutional collaboration network in ML-PCa research. Each node represents a research institution, with node size proportional to the number of publications. Colors denote distinct clusters of collaborating institutions, and connecting lines represent co-authorship links. **(C)** Cluster map of keyword co-occurrence for institutions. Each colored cluster corresponds to a major thematic area, with node size reflecting keyword frequency and link strength indicating co-occurrence. **(D)** Institutional citation clustering network. Nodes represent institutions, sized by total citation counts, and colored by citation clusters; link thickness reflects citation relationships.

Global institutional engagement in ML-PCa research spans 3,638 unique organizations. Annual publication trends demonstrate markedly accelerated output after 2015, with particularly steep growth emerging post-2020 (**Figure 4A**). Leading institutions by publication volume include the Chinese Academy of Sciences (42 publications), University of British Columbia (32 publications), and Shanghai Jiao Tong University (25 publications). Disparities emerge when assessing scholarly impact: while the Chinese Academy of Sciences leads in volume, its average citation rate (CPP=38.31) trails behind several Western counterparts. The University of Toronto achieves the highest CPP (70.54), followed closely by Case Western Reserve University (69.86) and University of Pennsylvania (59.83), signaling exceptionally influential research from these institutions (**Table 3**).

**Table 3:** Top 10 most productive institutions in ML-PCa research, including citation count, average citations per article, and centrality.

Institution	Documents	Citations	Average citations	Centrality
Chinese Academy of Sciences	42	1609	38.31	0.04
University of British Columbia	32	1330	41.56	0.06
Shanghai Jiao Tong University	25	357	14.28	0.03
University of Pennsylvania	24	1436	59.83	0.05
University of Toronto	24	1693	70.54	0.03
Zhejiang University	23	451	19.61	0.01
Case Western Reserve University	21	1467	69.86	0.03
Stanford University	21	668	31.81	0.03
University of California, Los Angeles (UCLA)	21	533	25.38	0.00
Harvard Medical School	20	1145	57.25	0.02

The institutional collaboration network reveals distinct structural patterns (**Figure 4B**). Western institutions, particularly University of British Columbia (highest centrality=0.06), Stanford University, and University of Pennsylvania, function as primary global connectors with extensive international linkages. Major Chinese institutions including the Chinese Academy of Sciences and Shanghai Jiao Tong University participate actively in global networks while exhibiting stronger regional cohesion. Research specialization clusters show clear geographic alignment (**Figure 4C**): North American and European institutions demonstrate concentrated expertise in digital pathology and artificial intelligence applications, whereas Chinese counterparts show heightened focus on cell-free DNA analysis and medical imaging diagnostics.

Citation network analysis confirms a multipolar global impact landscape (**Figure 4D**). Both the Chinese Academy of Sciences and University of British Columbia anchor densely connected citation cores, with additional regional clusters emerging: Chinese institutions including Huazhong University of Science and Technology form a distinctive citation collective, while institutions from the United States and United Kingdom establish self-contained high-impact modules. This structural configuration reflects China’s transition from peripheral contributor to central knowledge producer, while Western institutions maintain leadership in specialized methodologies and collaborative infrastructure.

### Journal distribution and citation impact

Globally, ML-PCa research has been disseminated across 10,437 unique journals, with *Cancers* (82 publications), *Frontiers in Oncology* (70 publications), and *Scientific Reports* (52 publications) constituting the dominant outlets (**Table 4**). Analysis of scholarly impact reveals *Cancers* as the most cited journal (1,127 total citations), though *Medical Physics* demonstrates superior per-article influence (CPP=27.79) despite moderate output volume. Notably, *Cancers*, *Scientific Reports*, and *IEEE Access* share equivalent long-term impact metrics (H-index=17), indicating comparable dominance within the domain (**Table 4**).

**Table 4:** Top 10 most productive journals in ML-PCa research, including citation metrics and 2024 impact factors.

Journal	Documents	Citations	Average citations	Impact factor (2024)	H_index
Cancers	82	1127	15.67	4.4	17
Frontiers in Oncology	70	946	13.37	3.3	18
Scientific Reports	52	1111	21.37	3.9	17
Diagnostics	39	690	18.42	3.3	16
IEEE Access	33	557	17.22	3.6	13
Sensors	33	597	18.53	3.5	12
Applied Sciences-Basel	30	245	8.17	2.5	9
Plos One	29	769	26.52	2,6	13
Medical Physics	27	719	27.79	3.2	13
Frontiers in Genetics	22	273	14.58	2.8	8

**Figure 5:**
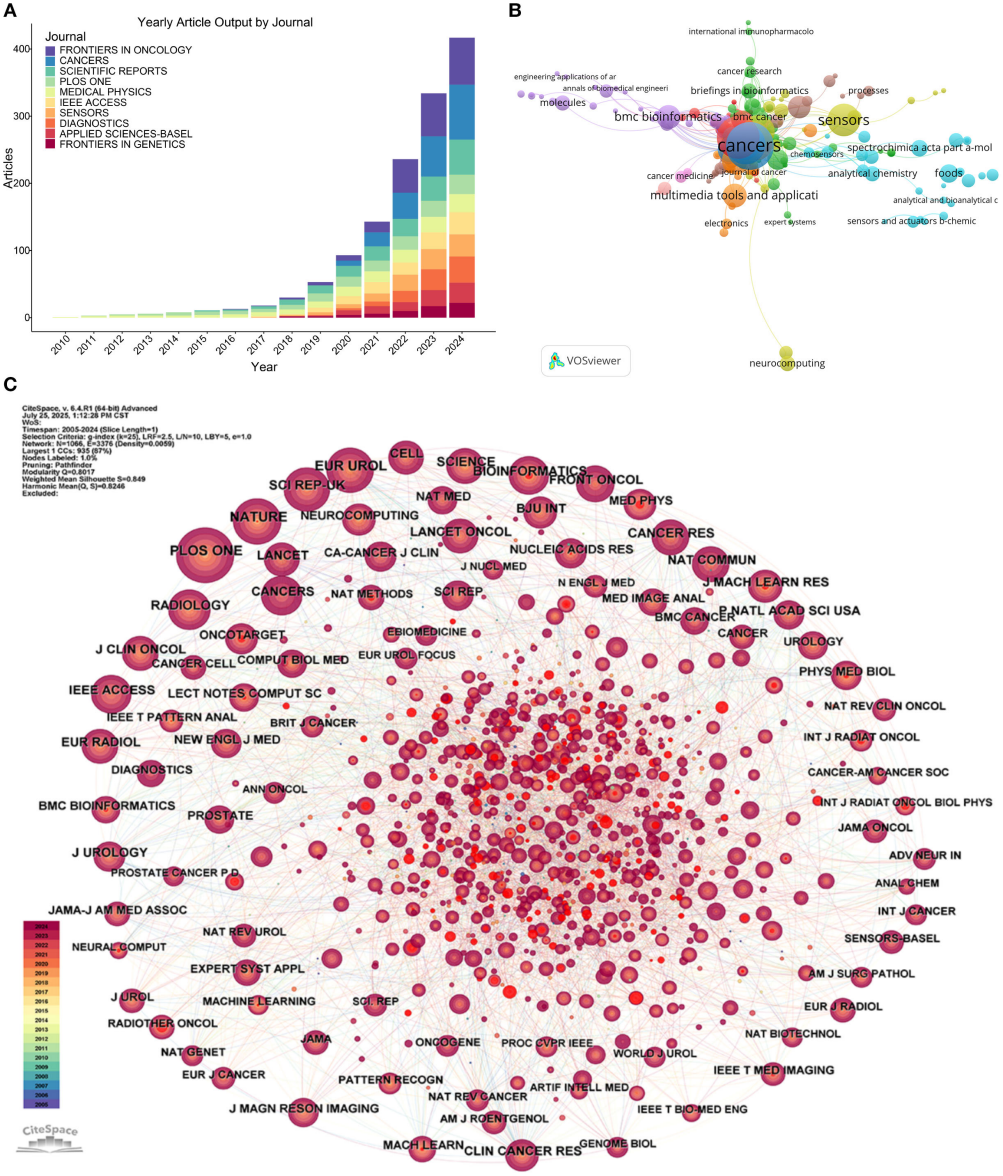
Journal-level bibliometric and thematic analysis of ML-PCa research. **(A)** Annual article output by top journals from 2010 to 2024, shown as a stacked bar chart where each color represents one of the leading journals. **(B)** Thematic clustering of journals based on their publication profiles in ML-PCa research: nodes represent journals, colored by dominant thematic cluster, with size proportional to publication volume. **(C)** Journal co-citation network: nodes represent journals sized by the number of times they are cited in ML-PCa articles, colors denote co-citation clusters, and edges indicate co-citation links.

Annual publication trends exhibit exponential growth after 2018, peaking in 2024 with *Cancers*, *Frontiers in Oncology*, and *Applied Sciences-Basel* leading this expansion (**Figure 5A**). Thematic clustering delineates distinct journal specializations: *Cancers* and *Frontiers in Oncology* anchor oncology-focused research, while *Sensors* and *IEEE Access* concentrate on computational modeling applications. *Medical Physics* emerges as a critical interdisciplinary hub through its bridging position between clinical and technological clusters (**Figure 5B**).

Co-citation network analysis positions high-impact journals including *Nature*, *IEEE Transactions on Medical Imaging*, and *Cancers* as foundational knowledge sources within tightly interconnected citation modules (**Figure 5C**). These citation relationships underscore the integration of machine learning methodologies into medical research paradigms. Temporal keyword evolution further reveals a transformative trajectory: pre-2015 research emphasized conventional techniques like feature extraction and support vector machines, whereas post-2020 publications increasingly prioritize deep learning, convolutional neural networks (CNN), and radiogenomics (**Supplementary Figure 1**).

**Figure 6:**
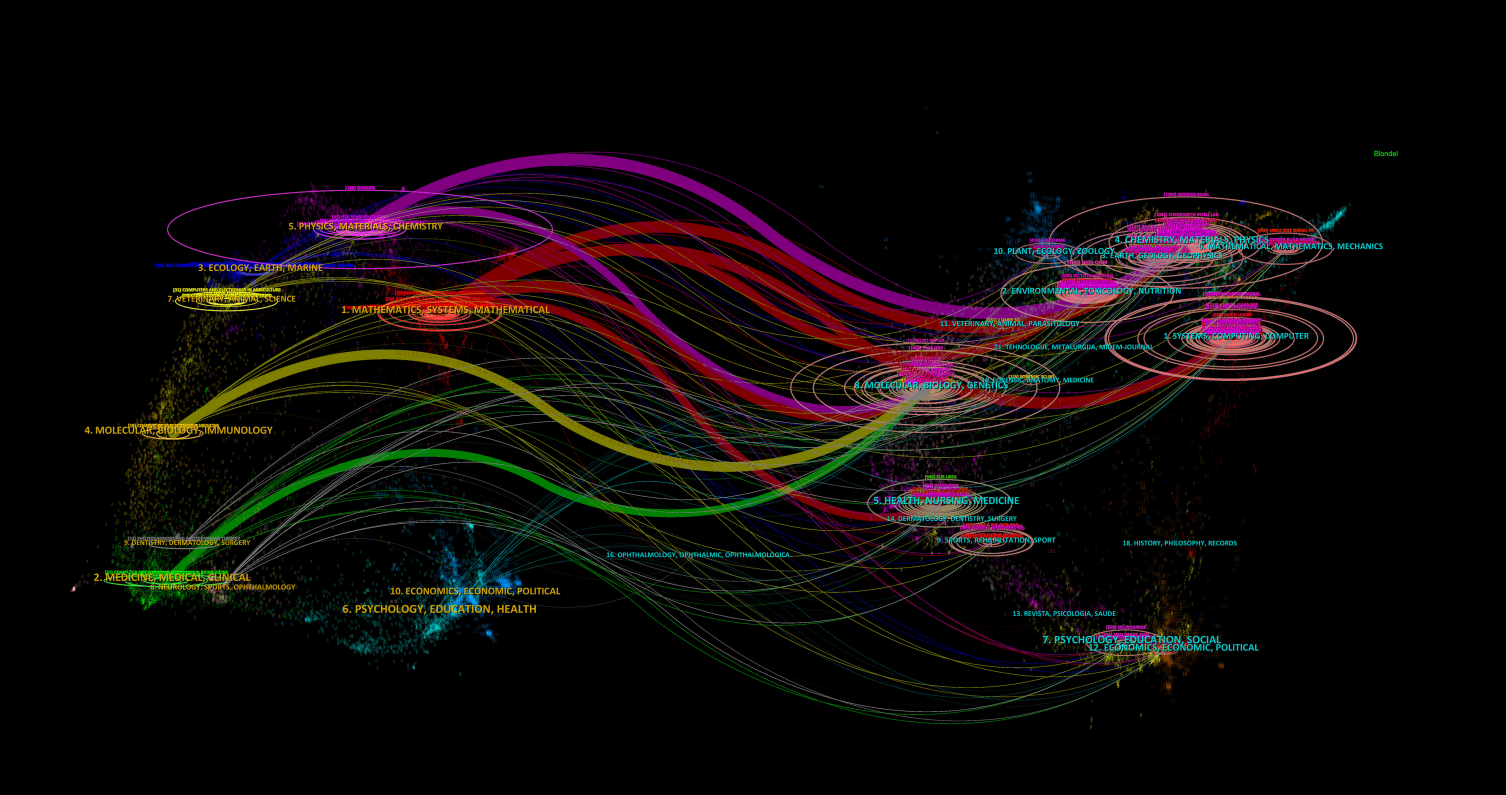
Dual-map overlay of citing and cited journals across scientific domains: left-hand map shows subject areas of citing journals, right-hand map shows domains of cited journals, and curved paths trace knowledge flows.

Analysis of cross-domain knowledge flows revealed three primary diffusion trajectories, each demonstrating distinct transdisciplinary pathways (**Figure 6**). The most prominent trajectory originates from systems and computer engineering journals, with knowledge subsequently adopted by publications in medicine and genetics research. A secondary pathway involves mathematical modeling sources transferring methodological innovations to clinical medical imaging applications. The third trajectory captures how sensor technology literature progressively informs molecular biology and immunology studies. Collectively, this tripartite diffusion pattern establishes ML-PCa research as a multidisciplinary convergence domain wherein computational innovations continually enable transformative advances in precision oncology, fundamentally reshaping biomedical discovery paradigms through cross-pollination of computational and life science methodologies.

### Author productivity and collaboration networks

**Figure 7:**
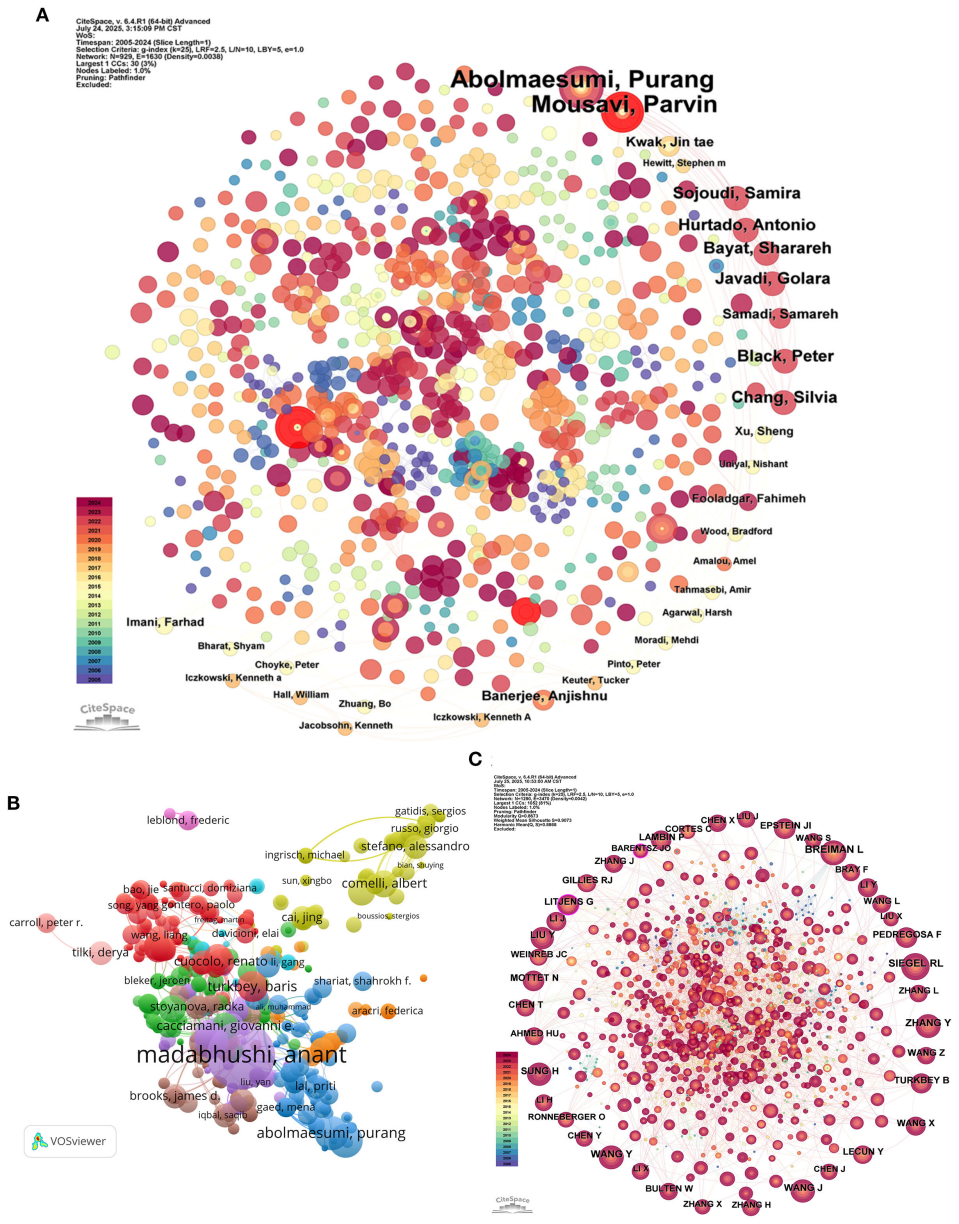
Author and topic-level mapping of ML-PCa research. **(A)** Publication productivity of authors in the current study: each node represents an author, node size reflects the number of articles they published. **(B)** Author citation network: nodes sized by citation counts of authors, edges represent citation relationships, indicating authors’ scholarly impact. **(C)** Cited-author co-citation network: nodes are authors cited in ML-PCa publications, sized by co-citation frequency, with colors denoting co-citation clusters.

The global ML-PCa research community comprises 12,345 authors, with 23.21% of publications involving international collaborations. As documented in **Table 5** and **Figure 7A**, Madabhushi Anant stands as the foremost contributor with 19 publications achieving 1,475 total citations, yielding an exceptional average citation rate of 77.63 per paper and an H-index of 16, establishing clear scholarly leadership. Additional high-impact authors include Abolmaesumi Purang and Mousavi Parvin (8 publications each), while specialists like Shiradkar Rakesh (average citations = 45.13) and Cuocolo Renato (47.50) demonstrate significant influence through focused high-quality output despite modest publication volumes.

**Table 5:** Top 10 most productive authors in ML-PCa research, including documents, citation count, average citations per article, and H-index.

Author	Documents	Citations	Average citations	H-index
Madabhushi Anant	19	1475	77.63	16
Abolmaesumi Purang	8	198	24.75	7
Mousavi Parvin	8	78	9.75	5
Shiradkar Rakesh	8	361	45.13	7
Turkbey Baris	7	199	28.43	8
Algohary Ahmad	6	216	36.00	5
Cacciamani Giovanni E.	6	170	28.33	5
Comelli Albert	6	128	21.33	5
Cuocolo Renato	6	285	47.50	6
Duddalwar Vinay	6	120	77.63	4

Collaboration network analysis reveals critical structural patterns (**Figure 7B**). Madabhushi Anant occupies the central network hub, maintaining robust ties with Abolmaesumi Purang, Comelli Albert, and Cacciamani Giovanni E., forming the nucleus of a densely interconnected cluster. Distinct multinational subgroups—notably Italian, Spanish, and U.S. researcher collectives—form peripheral subnetworks, reflecting the domain’s internationalized character.

Co-citation analysis identifies foundational knowledge contributors transcending direct publication output (**Figure 7C**; **Table 6**). Leo Breiman emerges as the most influential cited author (373 co-citations) with maximal centrality (0.09), underscoring his methodological primacy. Complementary authorities include Siegel Rebecca L. (327 citations) in epidemiological foundations and Wang Yuxing (252 citations) in statistical applications. Thematic clustering demarcates specialized knowledge streams: red and pink clusters concentrate on computational innovations in image recognition and deep learning, while blue and green clusters anchor clinical diagnostics and statistical epidemiology. Litjens Geert’s bridging centrality (0.13) despite moderate citations (202) confirms his role in cross-domain knowledge integration.

**Table 6:** Top 10 most cited-authors in ML-PCa research, including citation count and centrality.

Author	Citations	Centrality
Breiman Leo	373	0.09
Siegel Rebecca L.	327	0.01
Wang Yuxing	252	0.02
Zhang Yucheng	250	0.00
Wang Jing	239	0.00
Sung Hyuna	224	0.00
Liu Yuanbin	215	0.00
Pedregosa Fabian	205	0.00
Litjens Geert	202	0.13
Epstein Jonathan I.	197	0.03

Longitudinal keyword evolution documents a profound methodological transition (**Supplementary Figure 2**). Pre-2015 research emphasized feature engineering techniques (“support vector machines”, “texture analysis”, “TRUS imaging”). During 2015–2020, focus shifted toward integrated approaches (“radiomics”, “multiparametric MRI”, “deep learning”). Post-2020 innovations feature advanced architectures (“transformer models”, “attention mechanisms”) and multimodal integration (“radiogenomics”). This trajectory delineates the field’s progression from manual feature extraction toward sophisticated multimodal AI frameworks, increasingly prioritizing automated pattern discovery and biological correlation.

### Keyword co-occurrence and thematic evolution

**Figure 8:**
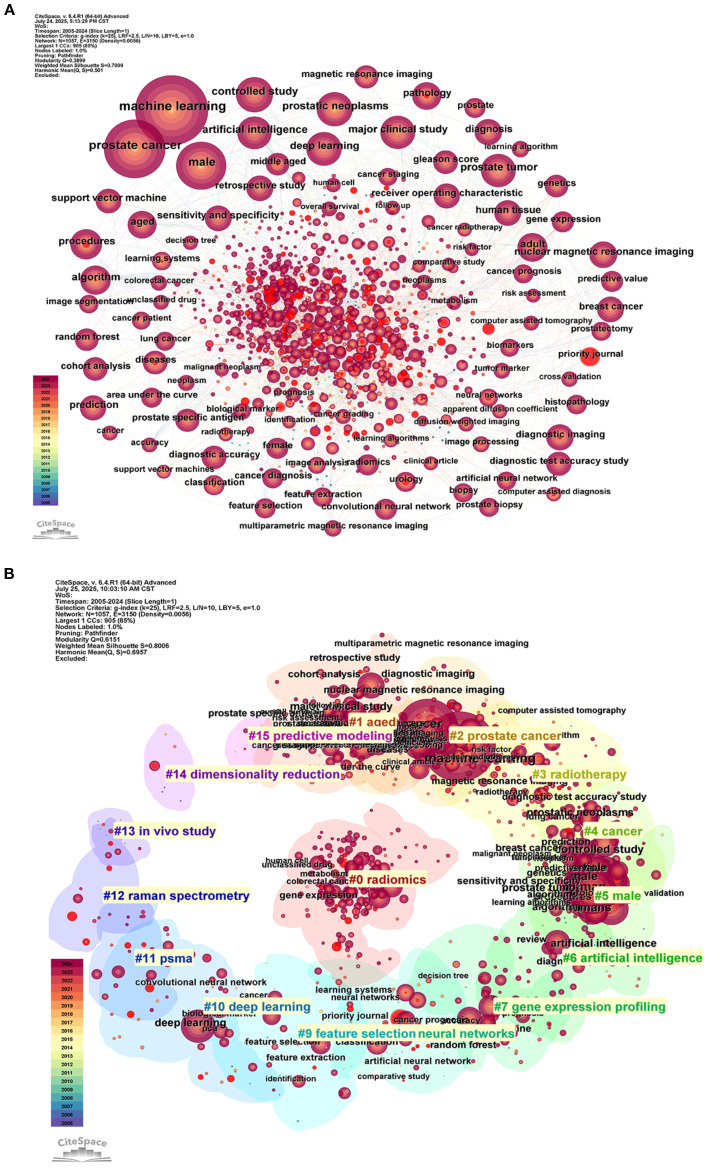
Keyword-based bibliometric analysis of ML-PCa research. **(A)** Co-occurrence network of author-assigned keywords: each node represents a keyword, node size reflects occurrence frequency, edges indicate co-occurrence relationships, and color intensity denotes citation centrality. **(B)** Keyword clustering map: Keywords are grouped into distinct thematic clusters based on co-occurrence, visualized with color-coded clusters. Node size represents frequency, and colors indicate cluster membership.

Keyword co-occurrence analysis establishes a multidisciplinary framework for ML-PCa research, integrating computational methods with clinical diagnostics (**Figure 8A**; **Table 7**). “machine learning” emerges as the dominant keyword (2,388 occurrences), followed by “prostate cancer” (1,726 occurrences), confirming their foundational importance. Centrality analysis identifies critical bridging terms including “Survival” (centrality=0.08), “System”, “Biopsy”, and “Validation” (centrality > 0.06), which facilitate knowledge exchange between technical and clinical domains. While less frequent, “Identification” and “MRI” also demonstrate significant network connectivity, underscoring their role in thematic integration.

**Table 7:** Top 25 keywords in ML-PCa research.

Keywords	Occurrences	Centrality
Machine Learning	2388	0.01
Prostate-Cancer	1726	0.02
Prediction	368	0.01
Diagnosis	363	0.03
Classification	278	0.01
Identification	117	0.00
Cancer	106	0.01
Expression	95	0.03
Risk	92	0.04
System	88	0.06
Model	86	0.02
Biopsy	76	0.06
Validation	73	0.07
Men	69	0.02
Features	67	0.05
MRI	61	0.03
Radical Prostatectomy	61	0.04
Images	59	0
Segmentation	59	0.01
Algorithm	57	0.04
Selection	56	0.01
Breast-Cancer	54	0.02
Feature-Selection	54	0.03
Survival	54	0.08
Carcinoma	43	0.01

Thematic mapping reveals distinct research concentrations through cluster analysis (**Figure 8B**). Key groupings include Cluster #0 (“deep learning”), Cluster #2 (“prostate cancer”), Cluster #6 (“artificial intelligence”), and Cluster #15 (“predictive modeling”), delineating core domains spanning diagnostic modeling and AI applications. Density visualization (**Supplementary Figure 3**) further highlights intensive research activity around “prostate cancer”, “diagnosis”, “biopsy”, and “identification”, forming the field’s substantive core.

Temporal evolution demonstrates a paradigm shift in research priorities (**Supplementary Figure 4**; **Supplementary Figure 5**). Early-phase research (pre-2015) emphasized conventional techniques like “pattern recognition”, “algorithm”, “classification”, and “support vector machine”. Post-2015 witnessed accelerating adoption of “deep learning”, “radiomics”, and “image segmentation”, with post-2020 research dominated by emergent concepts including “transfer learning”, “segmentation”, and “radiogenomics”. This progression reflects the field’s transition from feature engineering toward complex multimodal integration.

**Figure 9:**
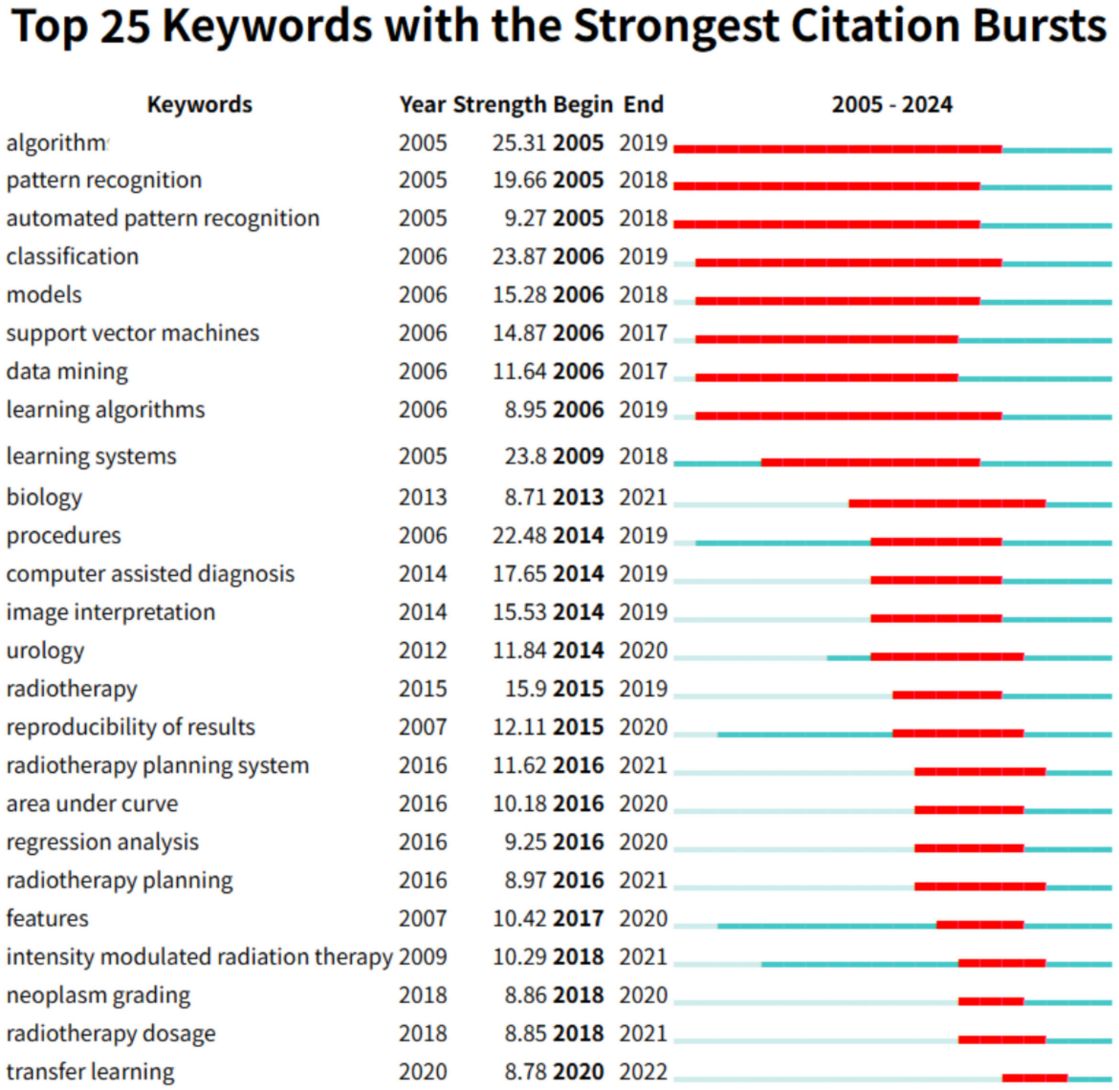
Top 25 keywords with the strongest citation bursts (2005–2024): red bars mark the active burst period for each keyword, and numbers indicate burst strength and timing.

Burst detection analysis (**Figure 9**) reinforces this evolutionary trajectory. Foundational methodologies including “algorithm” and “automated pattern recognition” exhibited strong bursts during 2005–2018, while contemporary emphases feature “transfer learning”, “radiotherapy dosage”, and “radiology” (2020–2024). This shift from methodological exploration to clinical implementation signifies ML-PCa’s maturation into a translational research domain focused on precision oncology applications.

To quantify the extent of clinically oriented research, we relied on bibliometric proxies. Author keyword frequency provided a conservative lower bound: validation appeared 73 times, representing approximately 2.8% of the corpus (73/2,632; **Table 7**). Keywords such as prospective, randomized, trial, implementation, or real-world were absent from the top 25, suggesting low prevalence overall.

### References and knowledge base

**Figure 10:**
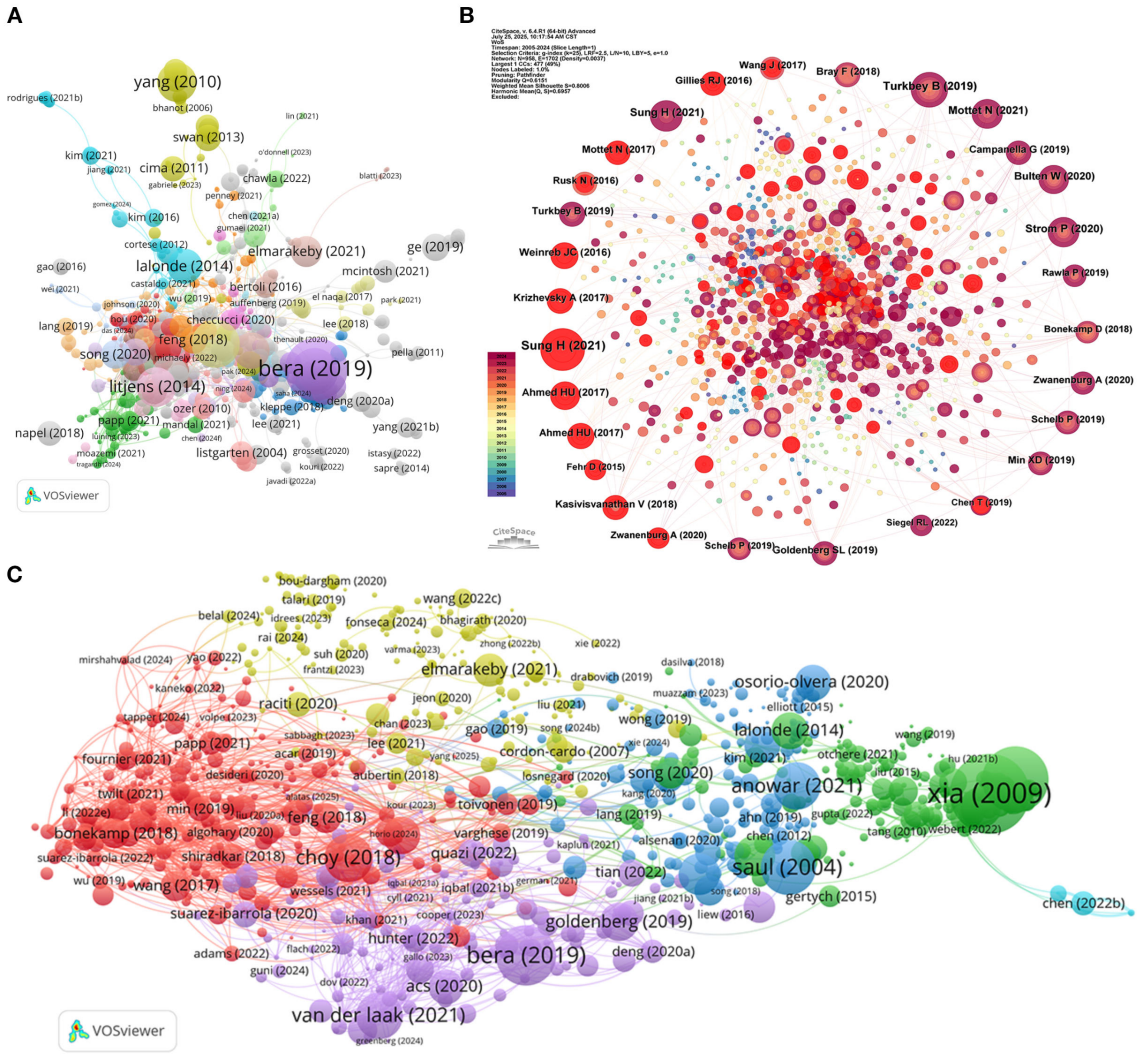
Reference-based bibliometric analysis in ML-PCa research. **(A)** Local co-citation network based on included literature: Constructed using VOSviewer, where each node represents a document, node size indicates the number of local citations, edges denote co-citation relationships between documents, and colors correspond to different thematic clusters. **(B)** Citation network analysis of external references cited by ML-PCa publications. Each node corresponds to a referenced article, with size proportional to the number of times it was cited. Nodes are color-coded by citation centrality. **(C)** Co-citation network of references. Nodes represent frequently co-cited references within ML-PCa literature. Clusters denote groups of papers that are often cited together, revealing major research themes and intellectual structure.

Core literature analysis identifies seminal works anchoring the intellectual structure of ML-PCa research. Bera et al. (2019) (23) (*Nature Reviews Clinical Oncology*, 880 citations) establishes AI’s transformative role in digital pathology and precision oncology, while Litjens (2014) (24), Fehr (2015) (25), and Lalonde (2014) (26) demonstrate foundational advances in MRI-based detection and tumor microenvironment analysis (**Figure 10A**; **Table 8**). These highly connected works reflect the domain’s emphasis on clinical-AI integration across radiology and pathology.

**Table 8:** Top 10 local-cited documents in the field of ML-PCa.

Authors	Year	Journal	Title	Citations
Bera et al. (23)	2019	Nature Reviews Clinical Oncology	Artificial Intelligence in Digital Pathology - New Tools for Diagnosis and Precision Oncology	880
Choy et al. (37)	2018	Radiology	Current Applications and Future Impact of Machine Learning in Radiology	532
Van der Laak et al. (44)	2021	Nature Medicine	Deep Learning in Histopathology: The Path to the Clinic	498
Litjens et al. (24)	2014	IEEE Transactions on Medical Imaging	Computer-Aided Detection of Prostate Cancer in MRI	362
Yang et al.	2010	Current Bioinformatics	A Review of Ensemble Methods in Bioinformatics	359
Goldenberg et al. (43)	2019	Nature Reviews Urology	A New Era: Artificial Intelligence and Machine Learning in Prostate Cancer	308
Fehr et al. (25)	2015	PNAS	Automatic Classification of Prostate Cancer Gleason Scores from Multiparametric Magnetic Resonance Images	303
Lalonde et al. (26)	2014	Lancet Oncology	Tumour Genomic and Microenvironmental Heterogeneity for Integrated Prediction of 5-Year Biochemical Recurrence of Prostate Cancer	279
Acs et al.	2020	Journal of Internal Medicine	Artificial Intelligence as the Next Step Towards Precision Pathology	248
Elmarakeby et al. (12)	2021	Nature	Biologically Informed Deep Neural Network for Prostate Cancer Discovery	225

The cited-reference network reveals central clinical guidelines underpinning ML-PCa development (**Figure 10B**; **Table 9**). Sung et al. (2021) (115 citations; **Table 9**) provides critical epidemiology data through GLOBOCAN 2020 (27), while Turkbey et al. (2019) (28)and Weinreb et al. (2016) (29) standardize mpMRI interpretation via PI-RADS v2.1/v2. Key validation studies – Ahmed et al. (2017)’s PROMIS trial (30) and Ström et al. (2020)’s AI-assisted biopsy system (31) – demonstrate the translational impact of these frameworks. Keyword clustering (**Supplementary Figure 6**) confirms this cross-disciplinary focus, with “machine learning,” “multiparametric MRI,” and “predictive models” forming dominant conceptual hubs.

**Table 9:** Top 10 cited references in the field of ML-PCa.

Authors	Year	Journal	Title	Citations
Sung H et al. (27)	2021	CA: A Cancer Journal for Clinicians	Global Cancer Statistics 2020: GLOBOCAN Estimates of Incidence and Mortality Worldwide for 36 Cancers in 185 Countries	115
Turkbey et al. (28)	2019	European Urology	Prostate Imaging Reporting and Data System Version 2.1: 2019 Update of Prostate Imaging Reporting and Data System Version 2	79
Mottet et al.	2020	European Urology	EAU-EANM-ESTRO-ESUR-SIOG Guidelines on Prostate Cancer-2020 Update. Part 1: Screening, Diagnosis, and Local Treatment with Curative Intent	56
Ström et al. (31)	2020	The Lancet Oncology	Artificial intelligence for diagnosis and grading of prostate cancer in biopsies: a population-based, diagnostic study	55
Bulten et al. (35)	2020	The Lancet Oncology	Automated deep-learning system for Gleason grading of prostate cancer using biopsies: a diagnostic study	54
Ahmed et al. (30)	2017	The Lancet	Diagnostic accuracy of multi-parametric MRI and TRUS biopsy in prostate cancer (PROMIS): a paired validating confirmatory study	48
Weinreb et al. (29)	2016	European Urology	PI-RADS Prostate Imaging – Reporting and Data System: 2015, Version 2	45
Krizhevsky et al.	2017	Communications of the ACM	ImageNet classification with deep convolutional neural networks	41
Campanella et al.	2019	Nature Medicine	Clinical-grade computational pathology using weakly supervised deep learning on whole slide images	40
Kasivisvanathan et.al (36).	2018	The New England Journal of Medicine	MRI-Targeted or Standard Biopsy for Prostate-Cancer Diagnosis	40

Co-citation analysis reveals five interconnected thematic clusters that define the methodological architecture of ML-PCa research (**Figure 10C**; **Table 10**). The red cluster anchors the field in algorithmic foundations, dominated by Pedregosa et al. (2011)’s Scikit-learn framework (126 co-citations) (32) and Chen et al. (2016)’s XGBoost model (33), establishing Python-based machine learning workflows as standard practice. Directly adjacent, the blue cluster encompasses clinical and imaging standardization, integrating epidemiological benchmarks (27) such as Sung et al. (2021)’s global cancer statistics with PI-RADS validation studies that operationalize mpMRI interpretation guidelines. Complementing these clinical pillars, the purple cluster develops radiomics frameworks for quantitative imaging biomarker extraction, exemplified by Gillies et al. (2016)’s (34) seminal work positioning medical images as mineable data sources. Parallel advances in bioinformatics methods (green cluster) underpin multi-omics data integration through gene expression analysis tools like GSEA and Limma, while the yellow cluster traces clinical translation pathways that bridge technical innovations with implementation workflows, ultimately connecting algorithm development to diagnostic applications.

**Table 10:** Top 10 co-cited references in the field of ML-PCa.

Authors	Year	Journal	Title	Co-citations
Pedregosa F et al. (32)	2011	Journal of Machine Learning Research	Scikit−learn: Machine Learning in Python	126
Breiman L et al.	2001	Machine Learning	Random Forests	121
Sung H et al. (27)	2021	CA: A Cancer Journal for Clinicians	Global Cancer Statistics 2020: GLOBOCAN Estimates of Incidence and Mortality Worldwide for 36 Cancers in 185 Countries	116
Weinreb JC et al. (29)	2016	European Urology	PI−RADS Prostate Imaging – Reporting and Data System: 2015, Version 2.1	83
Gillies RJ et al. (34)	2016	Radiology	Radiomics: Images Are More than Pictures, They Are Data	81
Turkbey B et al. (28)	2019	European Urology	Prostate Imaging Reporting and Data System Version 2.1: 2019 Update	79
Ahmed HU et al. (30)	2017	The Lancet	Diagnostic Accuracy of Multi−parametric MRI and TRUS Biopsy in Prostate Cancer (PROMIS)	78
Chen TQ et al. (33)	2016	KDD ‘16: ACM SIGKDD International Conference Proceedings	XGBoost: A Scalable Tree Boosting System	75
Cortes C et al.	1995	Machine Learning	Support−Vector Networks	75
Lambin P et al.	2012	European Journal of Cancer	Radiomics: extracting more information from medical images using advanced feature analysis	59

Highly cited and co-cited reference sets further reinforced this pattern. Landmark clinical validation studies (Strom 2020 (31); Bulten 2020 (35); Kasivisvanathan 2018 (36)) were represented, yet the majority of influential works emphasized methodological development, algorithmic benchmarking, or standardization (**Tables 9**, **10**). Taken together, these bibliometric signals converge on the conclusion that robust clinical validation and implementation studies constitute only a small minority within the field.

Bibliographic coupling (**Supplementary Figure 7**; **Table 11**) further validates research convergence into distinct domains: AI-driven clinical imaging applications (Choy et al., 2018 (37); Bera et al., 2019) (23), multi-omics integration platforms (Xia et al., 2009’s MetaboAnalyst) (38), and emerging multimodal fusion techniques (Liu et al., 2017’s Raman spectroscopy approach) (39).

**Table 11:** Top 10 bibliographic coupling references.

Authors	Year	Journal	Title	Citations
Xia et al. (38)	2009	Nucleic Acids Research	MetaboAnalyst: A Web Server for Metabolomic Data Analysis and Interpretation	1692
Marmion et al.	2009	Diversity and Distributions	Evaluation of Consensus Methods in Predictive Species Distribution Modelling	1095
Bera et al. (23)	2019	Nature Reviews Clinical Oncology	Artificial Intelligence in Digital Pathology – New Tools for Diagnosis and Precision Oncology	880
Saul et al.	2004	Journal of Machine Learning Research	Think Globally, Fit Locally: Unsupervised Learning of Low Dimensional Manifolds	721
Choy et al. (37)	2018	Radiology	Current Applications and Future Impact of Machine Learning in Radiology	532
Anowar et al.	2021	Computer Science Review	Conceptual and Empirical Comparison of Dimensionality Reduction Algorithms	500
Van der Laak et al. (44)	2021	Nature Medicine	Deep Learning in Histopathology: The Path to the Clinic	498
Liu et al. (39)	2017	Analyst	Deep Convolutional Neural Networks for Raman Spectrum Recognition: A Unified Solution	367
Litjens et al. (24)	2014	IEEE Transactions on Medical Imaging	Computer-Aided Detection of Prostate Cancer in MRI	362
Yang et al.	2010	Current Bioinformatics	A Review of Ensemble Methods in Bioinformatics	359

**Figure 11:**
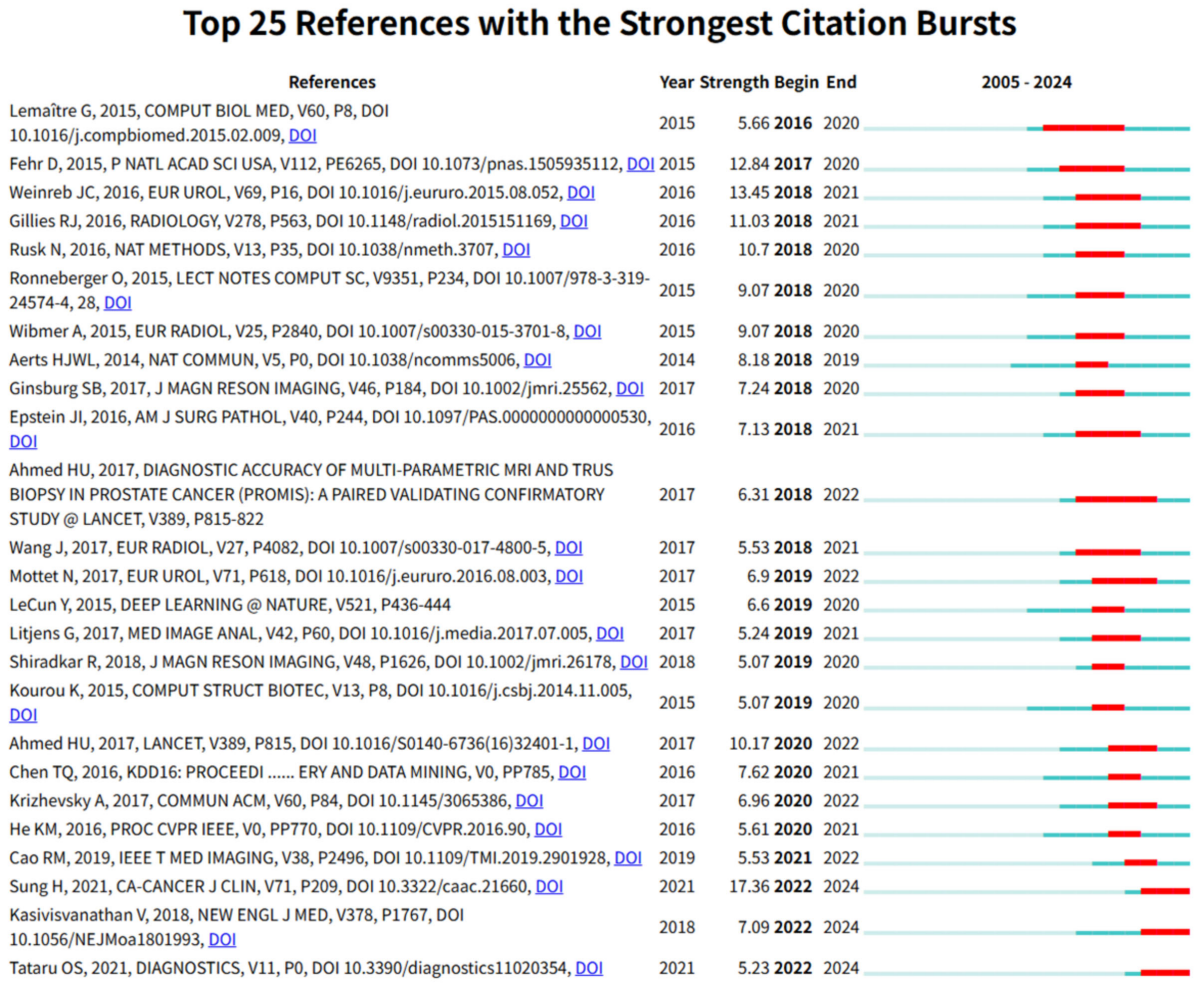
Top 25 references with the strongest citation bursts from 2005 to 2024. Red bars denote the active burst period for each reference, and the burst strength quantifies the sudden increase in citations over time, highlighting pivotal or trending literature in the field.

Temporal trends emerge through citation burst analysis (**Figure 11**). Early bursts (pre-2018) feature radiomics methods (Gillies et al., 2016) (34)and PI-RADS standardization (Weinreb et al., 2016) (29), while later surges prioritize clinical-AI integration: Sung et al. (2021)’s (27) epidemiology burst (intensity 17.36, 2022–2024) and Kasivisvanathan et al. (2018)’s validation of MRI-targeted biopsies (36). The 2018–2020 inflection saw deep learning methodologies (LeCun et al., 2015; XGBoost) (40) gain prominence, accelerating the shift toward clinically deployable tools.

## Discussion

The evolution of ML-PCa research represents a transformative convergence of computational innovation and precision oncology. These findings directly align with our study’s stated goals of not only mapping technological evolution but also critically examining the extent to which these advances have translated into clinical application. From its nascent interdisciplinary origins, the field has rapidly matured into a dynamic knowledge ecosystem characterized by accelerated global engagement, structural diversification across methodological and clinical domains, and reconfigured geopolitical knowledge hierarchies. This growth trajectory underscores profound synergies between algorithmic advancements and biomedical discovery, yet persistent translational gaps in clinical validation, interdisciplinary harmonization, and equitable implementation highlight critical challenges.

### Publication trajectory and intellectual emergence

The exponential growth in ML-PCa publications post-2015 signals a critical shift from theoretical exploration to clinical integration. This inflection aligns with pivotal advancements: deep learning maturation (CNN architectures enabling automated image analysis), data infrastructure proliferation (public repositories like PROSTATEx, TCIA), and cross-disciplinary consortia bridging computational and clinical domains (41, 42). The 82% output concentration in 2021–2024, coupled with 57,771 cumulative citations, underscores ML-PCa’s rapid maturation into a core oncology subfield. Yet, the recent citation dip (2023–2024) may reflect preliminary saturation in algorithm-focused studies, urging a pivot toward clinical validation and implementation research.

### Geopolitical dynamics and collaborative networks

China’s volumetric dominance (24.66%) highlights state-led investment in AI/healthcare priorities, though its lower MCP ratio (13.56%) suggests regional collaboration preferences. Conversely, the US’s centrality—despite smaller output (18.69%)—exemplifies global scientific integration (MCP ratio: 20.93%), reinforcing its role in knowledge diffusion. Germany’s outlier MCP ratio (39.29%) reflects strategic multilateralism within EU frameworks (Horizon Europe). The bipolar US-China collaboration hub (62 joint publications), alongside India’s autonomous citation cluster, reveals a stratified network topology. Risks include knowledge siloing (Western-centric clinical standards vs. Eastern imaging focus) and resource asymmetry, necessitating policies incentivizing Global South inclusion and data-sharing equity.

### Institutional output and impact asymmetries

Though Chinese institutions dominate publication volume (e.g., Chinese Academy of Sciences: 42 papers), Western counterparts lead influence (University of Toronto CPP: 70.54 vs. 38.31). This quantity-impact divergence stems from differential specialization: North American/European institutions focus on high-impact AI methodology and digital pathology (University of Pennsylvania’s radiomics innovations), while Chinese clusters prioritize imaging diagnostics. Western centrality in networks (UBC: 0.06 centrality) accelerates clinical translation, yet China’s emerging citation cores signify rising methodological credibility. Future gains necessitate bidirectional collaboration: integrating Chinese computational efficiency with Western clinical-validation pipelines.

### Journal integration and cross-domain synthesis

ML-PCa research exhibits robust interdisciplinary diffusion across three dominant pathways. Engineering-driven innovations (*IEEE Access*, *Sensors*) catalyze clinical adoption in oncology journals (*Cancers*, *Frontiers in Oncology*), while *Medical Physics* (CPP=27.79) bridges methodological and clinical domains with superior per-article influence. Temporal keyword evolution confirms deepening integration: post-2020 prioritization of “transformer models” and “radiogenomics” reflects the field’s shift from siloed applications toward biologically contextualized AI systems. Despite this convergence, fragmentation persists between computational and clinical clusters. Standardized reporting frameworks (MI-CLAIM) are urgently needed to streamline translation, particularly as *Scientific Reports* and *IEEE Access* emerge as critical venues for methodological prototyping preceding clinical validation.

### Author productivity and methodological evolution

Madabhushi Anant’s leadership (H-index: 16, CPP:77.63) epitomizes the blend of computational expertise and clinical partnerships driving high-impact innovation. Author clusters reveal globalized specialization: North American/European teams pioneer deep learning integration, while regional hubs (e.g., Italy’s Comelli cluster) refine clinical applications. Co-citation patterns affirm dual foundations: Breiman’s ML theory (centrality: 0.09) and Siegel’s epidemiological frameworks. Temporal keyword shifts—from SVM/feature engineering (pre-2015) to deep learning (2015–2020) and multimodal AI (post-2020)—highlight accelerating biological complexity. Future success hinges on nurturing “bilingual” researchers fluent in both biomedicine and algorithmic design.

### Thematic trajectories and translational bottlenecks

The evolution from technical exploration to clinical implementation defines ML-PCa’s maturation. Early emphases on “support vector machines” and “feature extraction” (pre-2015) transitioned toward integrative paradigms (“deep learning,” “multiparametric MRI”) during 2015–2020, culminating in today’s focus on multimodal frameworks (“attention mechanisms,” “radiogenomics”). Burst detection corroborates this trajectory: algorithm-centric bursts (2005–2018) gave way to clinical implementation keywords (“radiotherapy dosage,” burst intensity 5.72, 2020–2024). However, network centrality metrics expose critical translational gaps. High-connectivity terms like “validation” (centrality=0.06), “survival,” and “biopsy” remain underdeveloped compared to methodological terms, indicating insufficient linkage between AI performance metrics and clinical endpoints. Similar concerns have been noted in other bibliometric studies of AI in oncology, where methodological innovation often outpaces clinical integration (23, 43). For example, Elmarakeby et al. (12) highlighted that biologically informed neural networks demonstrated strong discovery potential, yet lacked systematic validation in prospective cohorts. This pattern mirrors our bibliometric evidence of an implementation gap. This imbalance—coupled with geographical bias in training data (78% Western cohorts)—hampers real-world deployment despite technical sophistication.

Our results are consistent with other domain-level bibliometric analyses, which have similarly observed rapid output growth coupled with translational inertia. For instance, recent mapping of AI in radiology underscored that fewer than 15% of studies incorporated prospective validation or multicenter trials, despite exponential publication growth (37, 44). By situating ML-PCa within this broader landscape, our analysis reinforces that bibliometric expansion alone is not a surrogate for clinical readiness.

### Knowledge foundations and future imperatives

Five interconnected thematic clusters underpin ML-PCa’s intellectual architecture. Algorithmic foundations (Scikit-learn, XGBoost) directly enable clinical-imaging standards (PI-RADS, PROMIS trials), while radiomics frameworks (Gillies et al.) and multi-omics integration tools (GSEA, Limma) support biologically anchored discovery. This convergence enables emerging translational pathways where methodologies evolve into clinical tools (Bera et al.’s digital pathology frameworks). Citation bursts confirm accelerating clinical emphasis: Sung et al.’s epidemiology (burst intensity 17.36, 2022–2024) and Kasivisvanathan’s biopsy trials dominate post-2020 citations. Yet significant voids persist in the knowledge base—fewer than 3% of highly cited works address ethical governance, health economics, or regulatory science.

Our bibliometric analysis is consistent with broader evidence syntheses: while publication volume and methodological innovation are expanding, high-quality clinical validation remains the exception rather than the rule. For instance, a systematic review of 41 ML RCTs found only a handful conducted with full adherence to CONSORT-AI (18); among 81 non-randomized deep learning imaging studies comparing AI with clinicians, only 9 were prospective and just six tested in clinical settings (45, 46); and in primary-care predictive algorithms, only 28% of FDA- or CE-marked tools satisfied even half of the Dutch AIPA guideline’s evidence criteria (47).

These external findings align closely with our internal bibliometric signals: validation appeared in just ~2.8% of articles (**Table 7**), trial-related terms were absent from high-frequency keywords, and clinical trial reports were under-represented in the most-cited clusters (**Tables 9**, **10**). Together, these patterns underscore a structural imbalance—abundant retrospective algorithmic benchmarks but scarce prospective, validated, and implemented studies.

Looking forward, closing this gap will require: (1) multi-institutional validation studies using standardized imaging biomarkers (48); (2) Federated Learning solutions for data-scarce populations (49, 50); and (3) SNOMED-CT integration to bridge EHR siloes (51). Without these, ML-PCa risks becoming a methodological echo chamber rather than a transformative clinical discipline.

### Research hotspots

#### Multimodal MRI deep learning diagnosis

Research in PCa diagnostics increasingly leverages multiparametric MRI (mpMRI)-based machine learning to improve detection, grading, and characterization (52). Deep learning models now integrate T2-weighted, diffusion-weighted imaging (DWI), and dynamic contrast-enhanced (DCE) sequences to enhance identification of clinically significant prostate cancer (csPCa) (53). For instance, the Deep Radiomics model, trained on 615 patients from four cohorts (PROSTATEx, Prostate158, PCaMAP, NTNU/St. Olavs Hospital), achieved a patient-level AUROC of 0.91 in independent testing, demonstrating robustness comparable to PI-RADS assessment (AUROC: 0.94) without significant difference (54). Similarly, an MRI-TRUS fusion 3D-UNet model tested on 3,110 patients showed superior sensitivity (80% vs. 73%) and lesion Dice coefficient (42% vs. 30%) over MRI-alone approaches, alongside higher specificity (88% vs. 78%) in 110 controls (55). These approaches provide more accurate clinical decision support than PI-RADS v2.0 alone.

#### CNN imaging feature engineering

CNNs excel in automatically learning discriminative features from prostate images, outperforming traditional handcrafted feature methods. Recent innovations include lightweight 3D-CNN variants (XmasNet, ResNet-based blocks) with transfer learning, enabling rapid convergence on small datasets; XmasNet achieved an AUC of 0.84 using 199 training and 200 test cases from PROSTATEx (56). Automated segmentation via nnU-Net followed by voxel-wise radiomics feature extraction and XGBoost classification balances interpretability and efficacy (54). Further enhancements integrate channel and spatial attention mechanisms to weight multiscale features, improving tumor boundary delineation and heterogeneity detection while increasing sensitivity by >5% (57).

#### Large-scale public datasets and shared platforms

Multicenter public datasets address single-institution limitations and establish validation benchmarks. The SPIE-AAPM-NCI Prostate MR Classification Challenge (PROSTATEx) provided 330 training and 208 testing lesions with standardized mpMRI quality control, while its successor PROSTATEx-2 focused on Gleason grade prediction (42, 58). The TCIA Prostate-MRI-US-Biopsy dataset (1,151 patients) has been extensively validated in >17 core publications Natarajan et al. Combined analysis of six independent microarray datasets identified high-confidence biomarker gene sets, significantly improving cross-cohort generalization and enabling multi-omics integration (42, 59).

#### Challenge-driven interdisciplinary collaboration

Public competitions foster synergy among clinical, physics, and computational experts to accelerate translation. Initiatives like the SPIE-AAPM-NCI PROSTATEx Challenge (launched 2016) catalyzed algorithm innovation with real-time validation at SPIE conferences SPIE (58, 60). The MVP-CHAMPION project integrates clinical, genomic, and imaging data within the Million Veteran Program, enabling closed-loop refinement of ML-PCa models for clinical deployment. Open-science platforms (e.g., Grand-Challenge.org) share preprocessing scripts, model code, and visualization tools, establishing transparent, reproducible community standards (61, 62).

### Overcoming the translational gap: future opportunities

ML holds transformative potential across prostate cancer research. In diagnostics, integrating multi-omics data (genomic, proteomic, metabolic) with high-resolution imaging enables identification of novel biomarker combinations, significantly enhancing early detection sensitivity and specificity (63). Therapeutically, ML facilitates personalized treatment by predicting optimal drug combinations/sequences based on patient genetics, tumor microenvironment, and treatment history—improving efficacy while reducing adverse effects (64). It further refines radiotherapy planning through precision dose delivery tailored to tumor morphology, maximizing therapeutic impact while sparing healthy tissue (15). Future advances center on enhanced data processing and modeling: Automated AI-driven data annotation (deep learning for pathology images) will streamline workflows (65), ensemble and transfer learning will boost model robustness and accelerate task-specific adaptation (66), and the development of explicable models remains critical for clinical trust and adoption (67).

### Strengths and limitations of the study

This study offers a comprehensive analysis of the rapidly growing field of ML in PCa, identifying key trends, influential authors, journals, and research hotspots. By utilizing multiple bibliometric tools, this study provides a multidimensional view of the field. However, the study’s reliance on bibliometric data introduces certain limitations, particularly in evaluating research quality beyond citation metrics. Furthermore, language bias and self-citation may introduce potential sources of errors in the data. Finally, while bibliometrics provide valuable insights into publication trends, they do not offer a nuanced understanding of the methodologies and clinical applicability of this study.

## Conclusion

This study reveals explosive growth in ML-PCa research, dominated by contributions from China and the United States. Key advances center on multimodal imaging integration, deep learning-driven tumor classification, and large-scale dataset utilization, offering transformative potential for early diagnosis and personalized therapy. However, critical barriers persist, particularly the limited proportion of studies progressing to clinical validation and real-world testing. This echoes prior systematic reviews of AI in cancer care that found less than one-third of published models underwent external or prospective validation (31, 35). Thus, despite methodological sophistication, clinical adoption remains the exception rather than the rule. Encouragingly, recent international initiatives such as the PI-CAI challenge and consortium have begun to directly address these barriers by fostering cross-population validation, methodological standardization, and benchmarking, with several prostate AI spin-offs already benefiting from this framework. Future progress hinges on establishing international consortia for validation studies, developing explainable AI systems, and creating open-access data repositories to accelerate clinical translation and optimize global prostate cancer management.

## Data Availability

The original contributions presented in the study are included in the article/**Supplementary Material**. Further inquiries can be directed to the corresponding authors.
