# Supplementary material for: Algorithms on the rise: a machine learning–driven survey of prostate cancer literature
Source: Front Oncol. 2025 Oct 2;15:1675459. doi: 10.3389/fonc.2025.1675459 (PMC12527887; doi:10.3389/fonc.2025.1675459)
Supplement: Supplementary file 1 [file DataSheet1.docx]

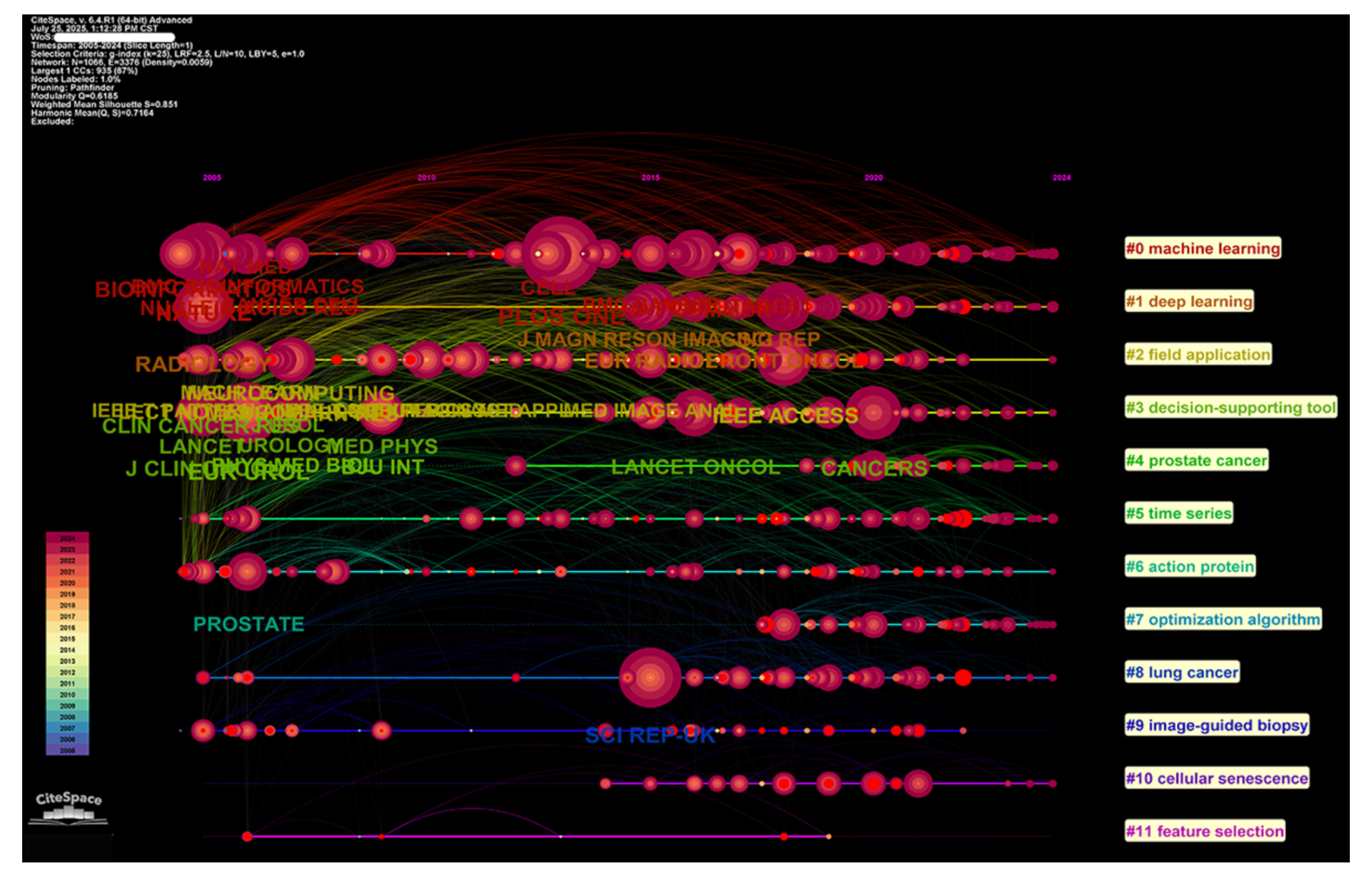


Supplementary Figure 1. Temporal keyword co-occurrence clustering for cited journals: a timeline layout showing the evolution of keyword clusters within the journals cited by ML-PCa publications; node size reflects keyword frequency and colors distinguish clusters.


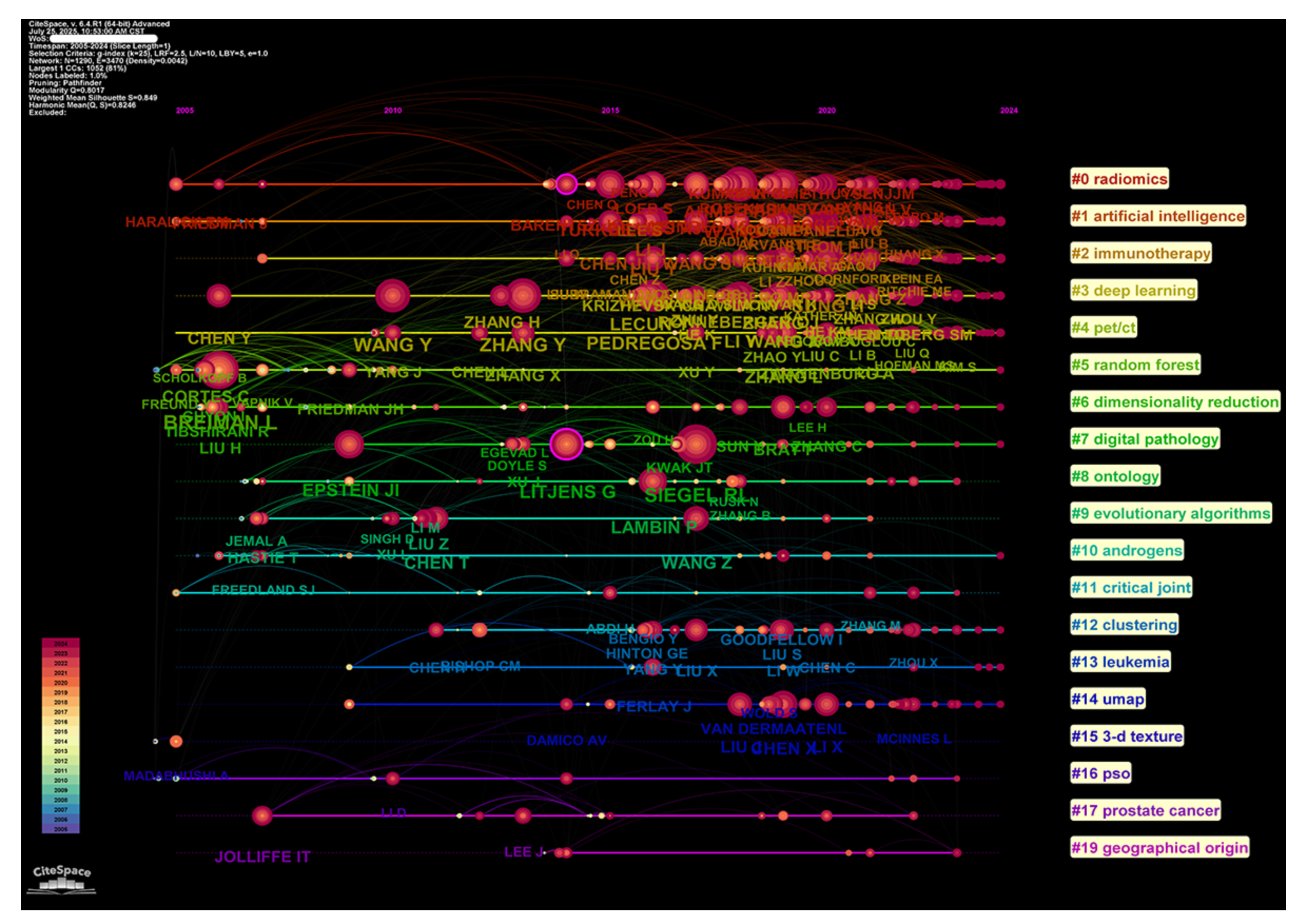


Supplementary Figure 2. Temporal clustering of keywords for cited authors: timeline view showing evolution of keyword clusters associated with cited-author works; node size indicates keyword frequency and colors distinguish clusters.


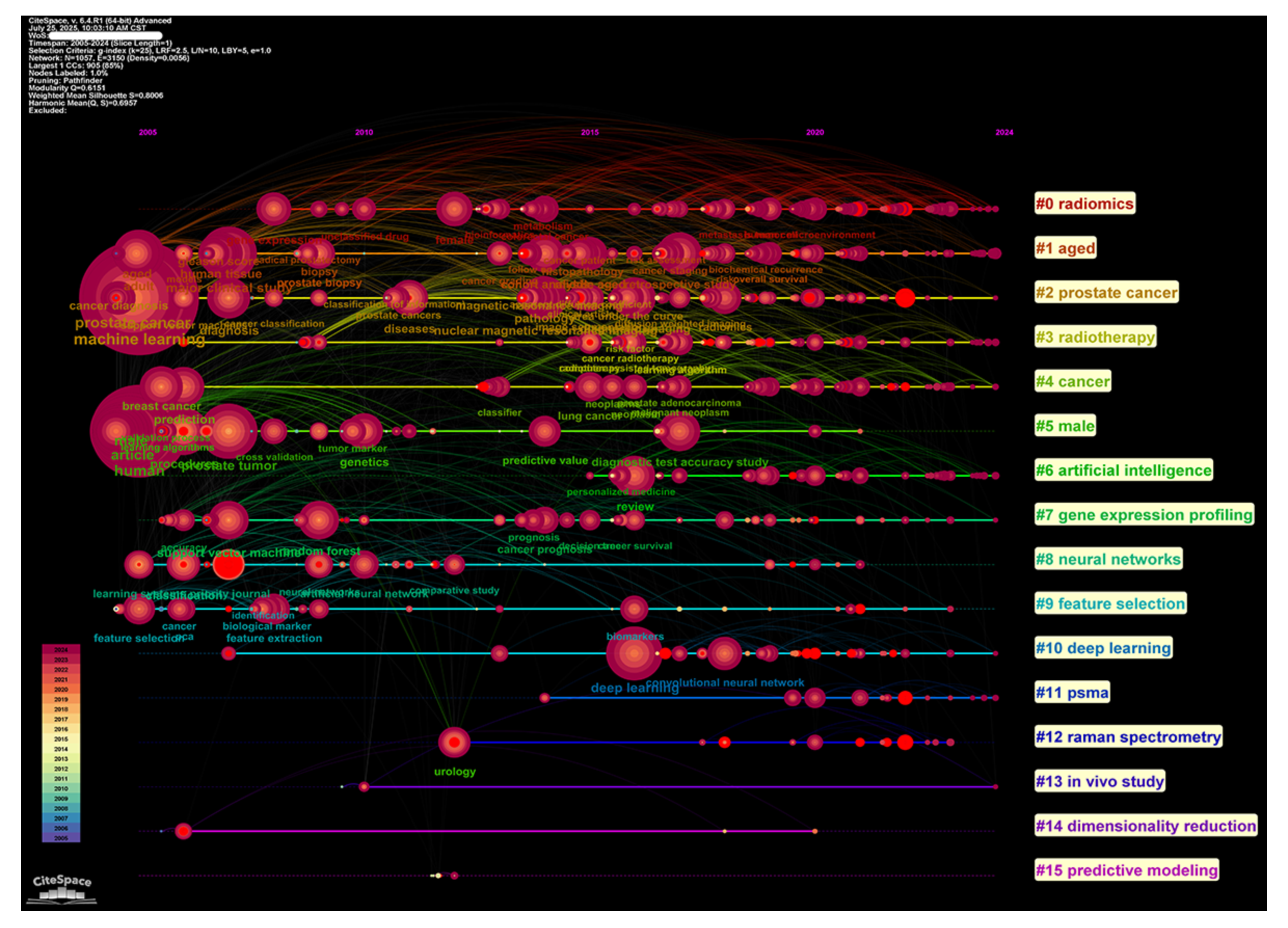


Supplementary Figure 3. Temporal overlay map of keyword usage: color gradient shows the average publication year for each keyword (blue = earlier, yellow = more recent), with node size indicating frequency.


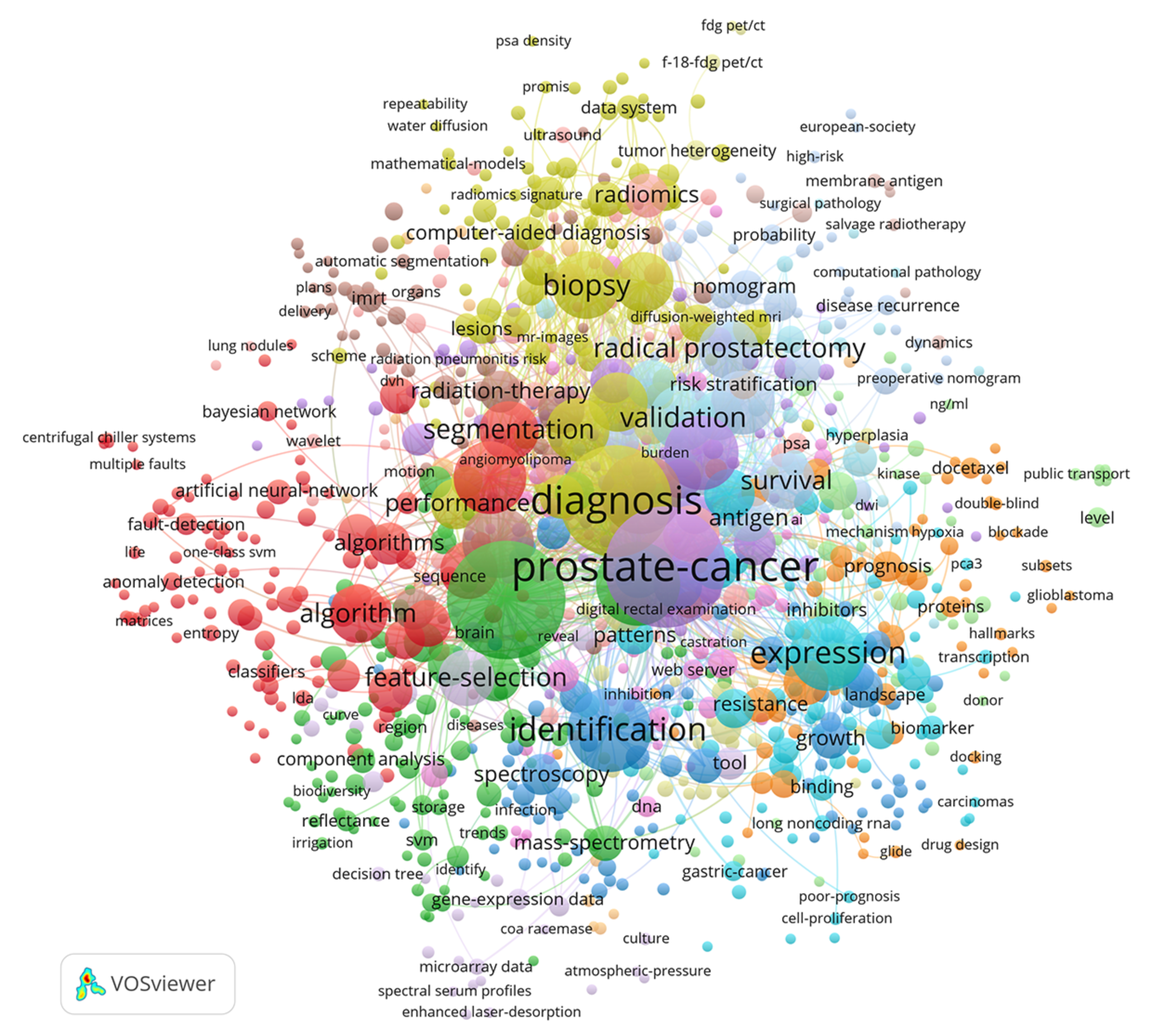


Supplementary Figure 4. VOSviewer keyword density map: highlights areas of high keyword concentration across the field, where warmer colors and larger nodes indicate higher density and frequency.


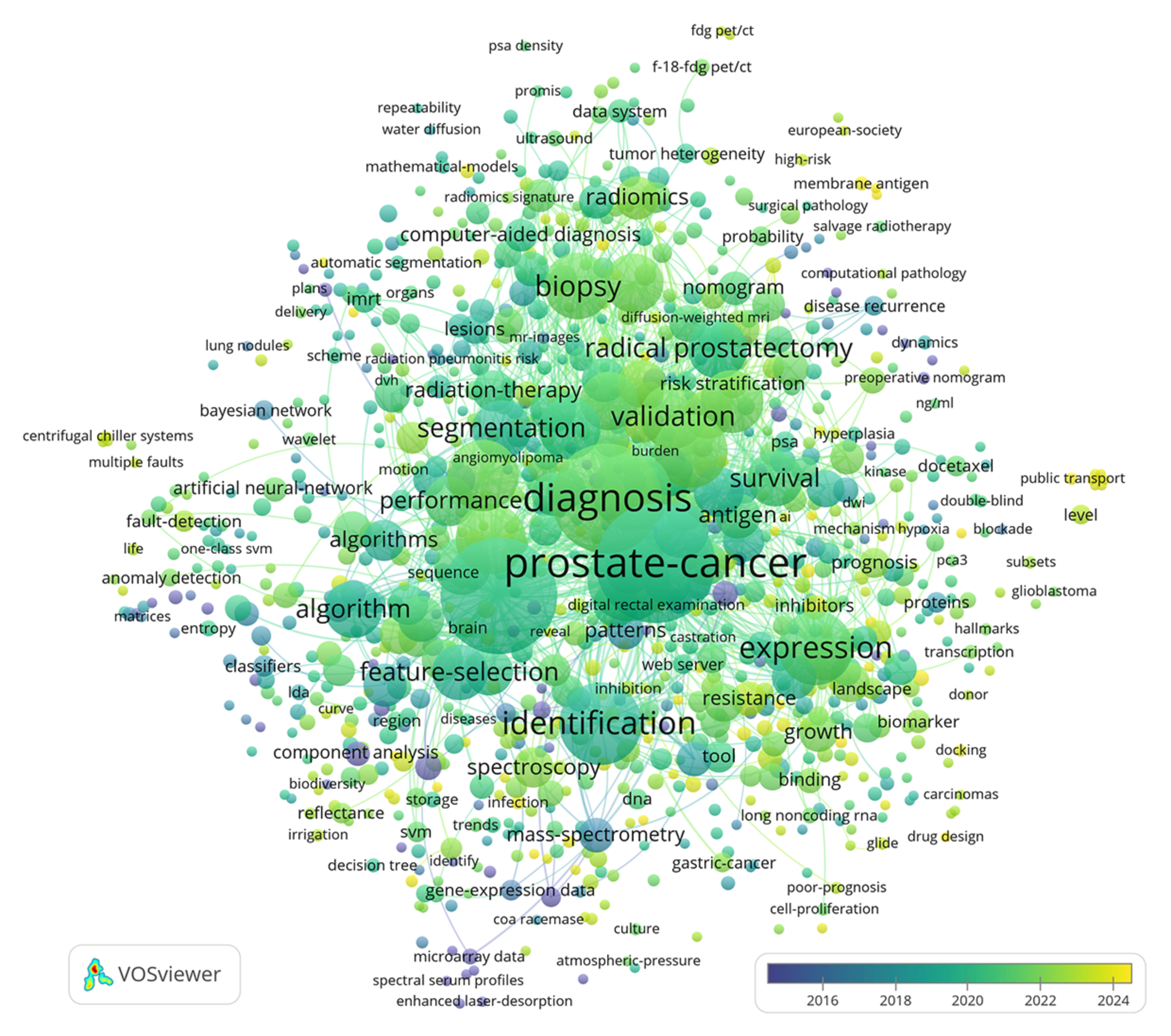


Supplementary Figure 5. VOSviewer temporal keyword map: displays the evolution of keyword prominence over time, with node color indicating publication year and size reflecting frequency.


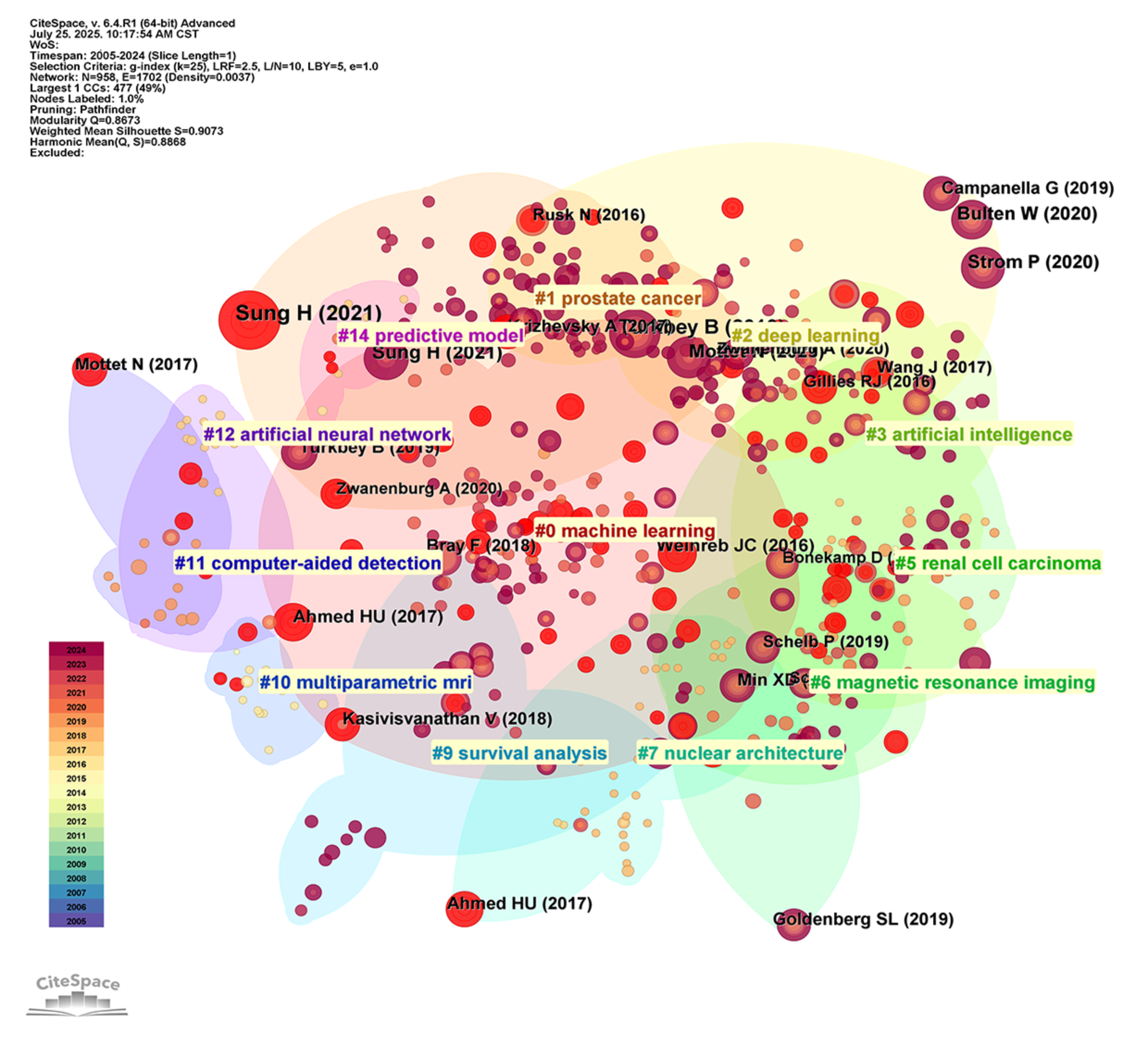


Supplementary Figure 6. Keyword clustering of cited references based on bibliographic data. Nodes represent cited references, grouped into thematic clusters using keyword co-occurrence. Colors indicate cluster membership, and node size reflects citation frequency.


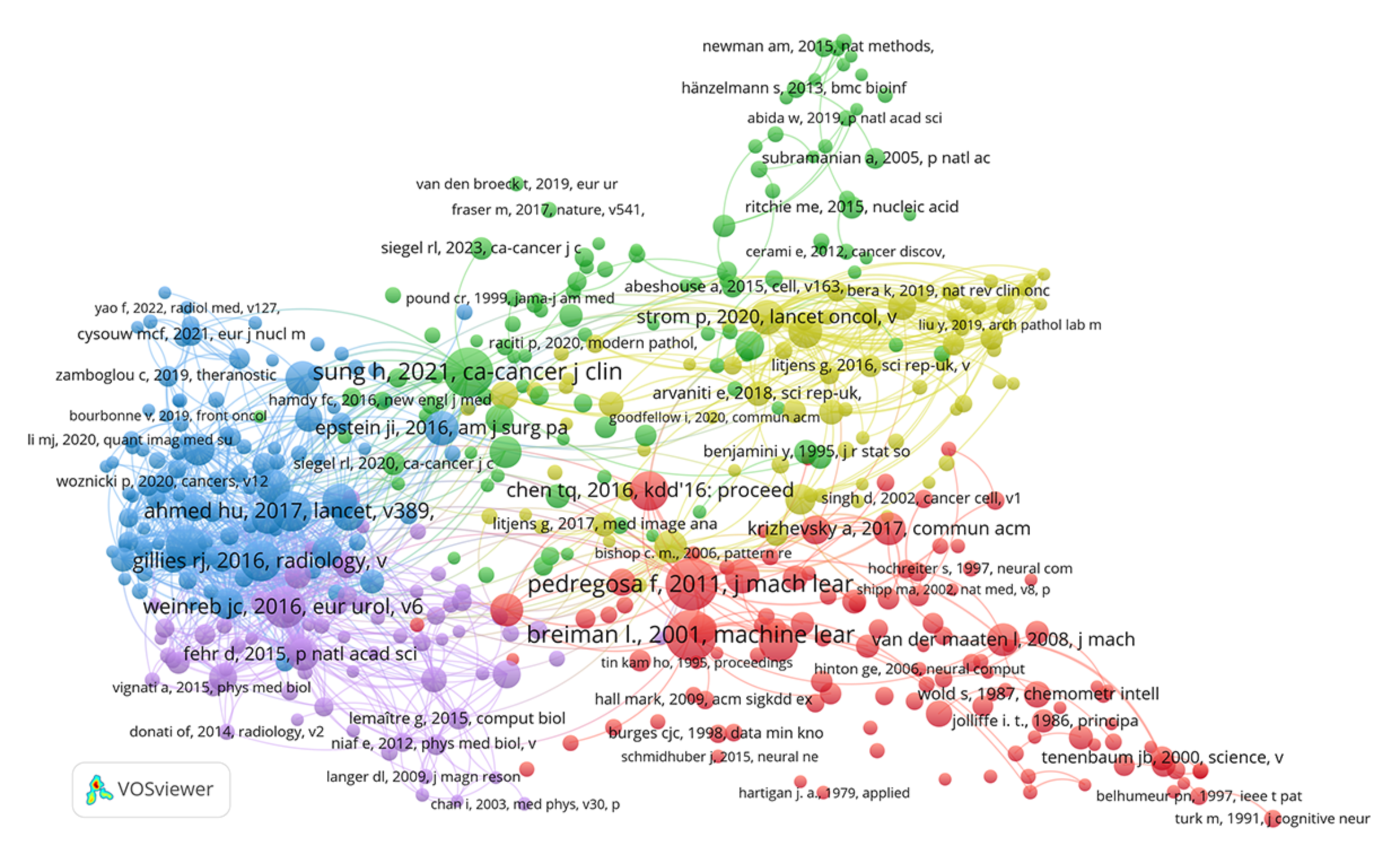


Supplementary Figure 7. Bibliographic coupling analysis. This map shows clusters of publications that share common references. Node size represents coupling strength (i.e., the number of shared citations), and color indicates cluster grouping.
